# Organic–Inorganic Multilayer Microcarriers with Superior Mechanical Properties for Potential Active Delivery in Fast-Moving Consumer Goods

**DOI:** 10.1021/acs.iecr.4c04503

**Published:** 2025-02-20

**Authors:** Daniele Baiocco, Benjamin T. Lobel, Mohammed Al-Sharabi, Olivier J. Cayre, Alexander F. Routh, Zhibing Zhang

**Affiliations:** † School of Chemical Engineering, University of Birmingham, Birmingham B15 2TT, U.K.; ‡ School of Chemical and Process Engineering, University of Leeds, Leeds LS2 9JT, U.K.; § School of Mathematics, Statistics, Chemistry and Physics, Murdoch University, Murdoch, Western Australia 6150, Australia; ∥ Department of Chemical Engineering and Biotechnology, University of Cambridge, Cambridge CB3 0AS, U.K.

## Abstract

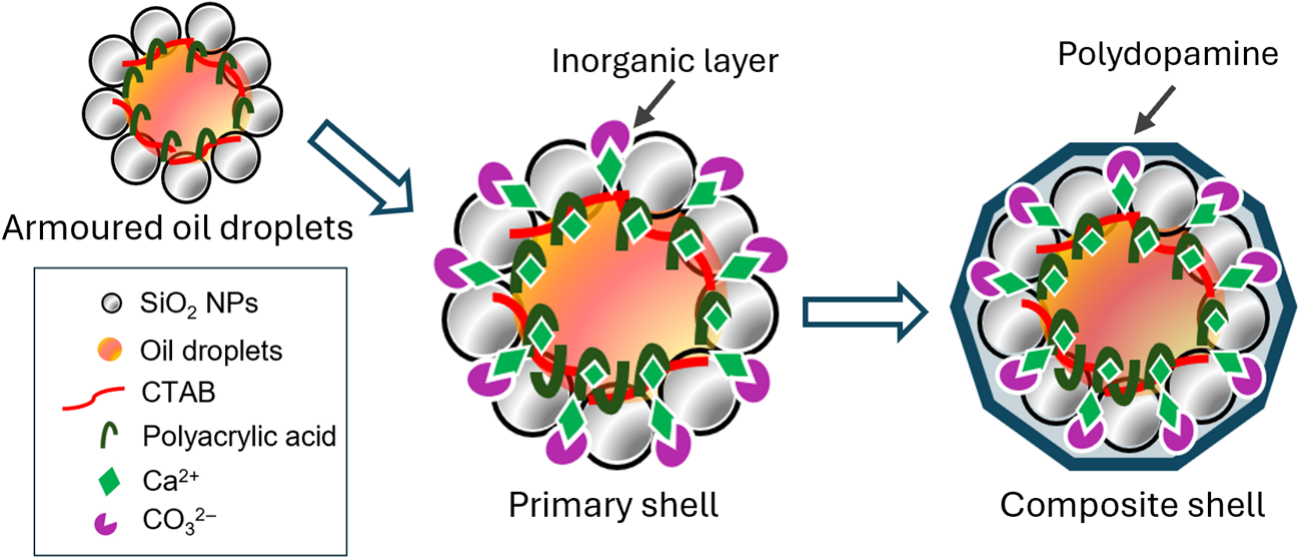

This study introduces an eco-friendly approach to fabricating superstrong, core–shell, composite microcapsules, offering a sustainable alternative to traditional insoluble microplastic-based materials like melamine-formaldehyde. These microcapsules were engineered with a thick CaCO_3_ shell formed via crystal ripening in the presence of water-soluble poly(acrylic acid), encasing a hexylsalicylate oil core armored by hydrophilic SiO_2_ nanoparticles. An additional polydopamine layer was deposited via oxidative autopolymerization at pH 8.5 for improved structural and surface properties of the resulting microcapsules. These microcapsules (*D*_3,2_ = 8.8 ± 0.3 μm) were spherical, with a relatively smooth surface, and exhibited unique mechanical properties, which are essential to broaden their applications in industry. Remarkably, compression tests showed a mean rupture stress of 73.5 ± 5.0 MPa, which dramatically surpasses any other inorganic/synthetic microcarrier reported in the literature. In addition, only 10–20% of the core active was released within 2 h into a mixed water-propanol medium used as an accelerated release test, where the solubility of the active oil is high, with full release over 3 days. Herein, we also propose a novel pathway-specific binding constant (PSBC) that describes the strong interaction between Ca^2+^ ions and poly(acrylic acid), in connection with their stoichiometric ratio. Overall, these microcapsules hold promise for multiple fast-moving consumer goods, where maximizing the mechanical strength of microcapsules for encapsulation of valuable functional actives is paramount; this includes but is not limited to energy storage, household, agrochemical, personal care, and healthcare applications.

## 1 Introduction

Many fragrances, flavors, and bioactive compounds are immobilized within core–shell or matrix microcapsules across multiple industrial sectors, particularly in fast-moving consumer goods (FMCG).^1^ This approach offers numerous advantages, including enhanced stability to prolong shelf life of the final product by safeguarding valuable actives from harsh environmental stressors, such as pH fluctuations, and ultraviolet (UV) radiation,^2^ and controlled release. In addition to desirable barrier and surface properties, a critical focus of microencapsulation research is the mechanical properties of microcapsules, which directly influence their stability, durability, and overall performance in diverse applications such as detergents, foams, and cosmetics.^3^ Consequently, substantial efforts have been dedicated toward engineering shell materials tailored to meet specific mechanical requirements.^4^

Synthetic materials like polyurethane, polyurea, and polystyrene have emerged as popular choices for microcapsule shells due to their ability to provide a balance of chemical and mechanical resistance.^5^ While these materials can offer effective barriers for active ingredients, their nonbiodegradability poses concerns regarding environmental sustainability, contributing to the accumulation of microplastics in the environment.^6^

Metal–organic frameworks (MOFs) offer broad-spectrum properties in drug delivery,^7−9^ such as high surface area and chemical/thermal durability. However, their crisscrossed three-dimensional (3D) crystalline lattices are spongy and porous, often leading to intrinsic mechanical weakness.^10^ Environmentally benign inorganic materials, such as silica, offer distinct advantages in terms of thermal stability and chemical inertness, making them suitable for applications requiring temperature resistance. Yeom et al.^11^ fabricated core–shell silica microcapsules for fragrance (hexyl cinnamaldehyde) delivery. The shell was prepared on the surface of an oil-in-water emulsion template using tetraethyl orthosilicate (TEOS) initially dissolved in both the dispersed (hydrophobic) and continuous (hydrophilic) phases, across multiple stages. Although these microcapsules retained the fragrance for over 80 days in a harsh liquid environment containing anionic surfactant (15 wt % sodium dodecyl sulfate, pH 3.5) at 60 °C, their shell thickness was only 60–100 nm, which suggests their good barrier properties yet mechanical weakness.

Within industry, Cardoso et al.^12^ have patented a methodology for fabricating core–shell silica microcapsules laden with hydrophobic or hydrophilic actives. These microcapsules feature a continuous shell, potentially capable of segregating actives for periods compatible with various applications. However, the inherent brittleness and mesoporosity of silica render these shells susceptible to cracking or breaking under mechanical stress, which remain to be improved before they can be used for widespread commercial applications.

Calcium carbonate (CaCO_3_) has emerged as an effective alternative material to synthetic polymers for forming microcapsule shells. In addition to being environmentally benign, it is economically viable for large-scale production.^13^ Wang et al.^14^ proposed a simple and potentially scalable methodology for fabricating core–shell microcapsules, featuring a limonene core and a thick crystallized CaCO_3_ shell (1–2.5 μm). The encapsulated limonene exhibited a prolonged release in neutral pH water at 85 °C, whereas a burst release occurred at pH 2. While this technology has advanced scientific understanding, the applicability of these microcapsules may be limited in sectors where acidic and/or alcohol-based environments are required, such as ultraprocessed food (UPF), flavor-enriched beverages, and cosmetic formulations. Despite its great promise, the mechanical and barrier properties of calcium carbonate-based shells still require significant improvement for a broader range of commercial applications.^15^

When compared to organic materials, employing only calcium carbonate to form the microcapsule shell may lead to intrinsic porosity, significantly restricting its utilization in encapsulating small molecules.^15^ Long et al.^13^ reported an approach to mitigate this porosity by producing double-shell composite microcapsules, featuring ripened CaCO_3_ crystals as the outer shell and melamine-formaldehyde (MF) polymer as the inner shell. This design resulted in over 25 times less leakage of the core oil compared to CaCO_3_-only shells, due to the inner MF coating. Although effective, MF is not (bio)degradable. Alternatively, White et al.^16^ produced gold-shell microcapsules, featuring a nonleaky shell, capable of releasing their active load on-demand via focused ultrasounds. While promising, gold may be impractical for large scale applications due to its high costs. Consequently, there is an urgent need to evolve industrial activities toward cheaper and more environmentally benign methods, decreasing the use of unsustainable microplastics and/or expensive metals.^11,17^

In our recent study, we opened an avenue for developing environmentally conscious hybrid composite shells by combining inorganic (CaCO_3_) and organic (polydopamine) materials to create a two-layer shell.^18^ This yielded microcapsules with promising barrier and adhesive properties, under mimicked real-world settings. Although acceptable, the mechanical properties of these microcapsules (mean rupture stress 2.2 ± 0.3 MPa) should be improved in order to expand their potential applicability across a broader range of industrial sectors, where tougher microcapsules are required, such as those for encapsulating phase change materials, pharmaceuticals, agrochemicals, paints and lacquering agents.^2^

The application of readily degradable hydrogels, such as fungal chitosan, hyaluronic acid, and poly(acrylic acid) (PAA) to form matrix microcapsules and/or microneedles is hindered by their weak mechanical properties.^1,3,6,19,20^ Liu et al.^21^ produced mechanically stable PAA-coated polyimide substrates for underwater applications, with excellent adhesive properties onto inert substrates due to hydrogen bonding between the two species. However, their remarkable performance properties are attributed to the presence of polyimide, which thus remains nondegradable.^21^

Zhong et al.^22^ found that the mechanical properties of PAA hydrogels are significantly influenced by the content of both covalent and ionic cross-linkers, such as methylenebis(acrylamide) and Fe^3+^ ions.^22^ This implies that metal ions can electrostatically interact with the copious carboxyl groups along the backbone of oppositely charged polymers, like PAA, improving their mechanical behavior. Despite the formation of hydrogels with remarkable mechanical properties, iron is a heavy metal, which may represent a drawback to many commercial applications. Similar considerations were drawn by Fu et al.^23^ who developed mesoporous PAA-calcium phosphate nanoparticles for biomedical applications.

Although promising, to the best of the authors’ knowledge, composite architectures, made of CaCO_3_ in the presence of PAA, have not been applied to encapsulation, such as core–shell fragrance/flavor encapsulation. Consequently, the present study aims to investigate the feasibility of developing environmentally benign core–shell microcapsules with enhanced mechanical properties. To this end, organic–inorganic composite architectures are engineered, featuring a thick inorganic shell (CaCO_3_) developed in the presence of PAA as a reinforcing agent to aid in the interfacial crystal ripening of CaCO_3_.^24^ An outer PDA wrapping is subsequently deposited onto the primary CaCO_3_–PAA microcapsules, to further enhance their mechanical stability, barrier and surface properties.^18^

Our findings suggest that the inorganic–organic architectures may offer a novel pathway to bolstering the mechanical, barrier and surface properties of microcapsules, toward more user- and environmentally conscious formulations.

## 2 Materials and Methods

### 2.1 Materials

Free-flowing calcium chloride (CaCl_2_) flakes (CAS 7440–70–2) were procured from Scientific Laboratory Supplies (SLS, Nottingham, U.K.). They were milled into a fine powder using a hand-held agate mortar (diameter ∼70 mm) and pestle. Anhydrous sodium carbonate (Na_2_CO_3_; CAS 497–19–8), poly(acrylic acid) solution (PAA, 25 wt %; ∼240 kDa; CAS, 9003–01–4), and sodium tetraborate (Na_2_B_4_O_7_; CAS 1330–43–4) buffer tablets (pH 9.2; makeup volume = 0.1 L/tablet) were supplied by Fisher Scientific (Loughborough, U.K.). Pristine hydrophilic fumed silica nanoparticles (SiO_2_ NPs) with an average particle diameter of <10 nm and specific surface area of ∼300 m^2^/g (Aerosil 300; CAS 7631–86–9) were sourced from Evonik Industries AG (Essen, Germany, EU). Dopamine hydrochloride (C_8_H_11_NO_2_·HCl; CAS 62–31–7) was provided by Alfa Aesar (Heysham, Lancashire, U.K.). All other chemicals were supplied by Merck (Gillingham, Dorset, U.K.). Specifically: hexyl salicylate (HS, >99.0%, specific density 1,040 kg·m^–3^; CAS 6259–76–3) as the model oil, Nile Red (NR; CAS 7385–67–3) as the fluorescence sensing dye, sorbitan trioleate (Span 85; CAS 26266–58–0) and polyoxyethylene sorbitan monooleate (Tween 80; CAS 9005–65–6) as emulsifiers, cetyltrimethylammonium bromide (CTAB; CAS 57–09–0) as a cationic surfactant, potassium bromide (KBr; CAS 7758–02–3) as an infrared (IR)-transparent window material, commercial CaCO_3_ powder (∼50 μm; CAS 471–34–1) as a control for Fourier transform infrared (FTIR) chemical analysis, fuming hydrochloric acid (HCl; 36% w/v; CAS 7647–01–0) and sodium hydroxide (NaOH; CAS 1310–73–2) as pH adjusters. Additionally, 1-propanol (PrOH; CAS 71–23–8) was used as the blank and receptor medium for UV–visible (UV–vis) analysis. Ethylene-diaminetetraacetic acid (EDTA; CAS 60–00–4), sodium bicarbonate (NaHCO_3_; CAS 144–55–8), and ethanol (CAS 64–17–5) served as cleansing agents. LR white acrylic resin (CAS 94188–59–7) was used to embed TEM samples. All chemicals were of analytical grade, stored according to their Safety Data Sheet (SDS) guidelines, and utilized without further purification. All solutions were prepared using Milli-Q water (18.2 MΩ·cm^–1^ at 25 °C).

### 2.2 Fabrication of Microcapsules with SiO_2_–PAA-CaCO_3_ Shells

Step 1: An aqueous suspension of pristine SiO_2_ NPs (0.8 g) was generated under continuous stirring (700 rpm; Rushton turbine Ø 34 mm; IKA Eurostar 20, Germany, EU) over 10 min to disperse the NPs in water (100 mL) within a double-glazed cylindrical vessel (capacity 500 mL; liquid height/tank diameter ∼1; clearance/impeller diameter ∼3/4) with four baffles (baffle width/tank diameter ∼0.1). The suspension was then titrated to pH 9.2 using a borate buffer.

Step 2: The core oil (HS: 3 g, HS weight: *w*_HS_) primed with fluorescence dye Nile Red (NR: ∼3 mg; NR weight: *w*_NR_; ∼0.1% *w*_NR_/*w*_HS_) was added to the suspension to generate oil-in-water (o/w) emulsion droplets under mechanical stirring (Ø 34 mm; 700 rpm; 10 min) leading to SiO_2_ NP-armored droplets (Step 2.1). At the same time, cationic CTAB (37.5 mg) at its critical micelle concentration (CMC ∼ 1 mM at 25 °C)^25^ was added under stirring, leading to a slight reduction in the pH to ∼8.9.

Step 3: 2.0 g of a 25% (w/w) PAA aqueous solution (*m*_PAA_ = 0.5 g) was instilled dropwise to the emulsion, under continuous stirring (700 rpm) to adsorb onto the positively charged oil–water interface, in the presence of SiO_2_ NPs and CTAB. The pH was maintained to ∼8.9, where PAA exhibited a strong negative charge (Supporting Information, Table S1 and Figure S1), remaining outside the higher solubility zone of SiO_2_.

Step 4: An aqueous suspension (50 mL; 1.0 M) of calcium chloride (CaCl_2_) was introduced into the stabilized emulsion at a controlled flow rate (200 μL min^–1^) using an infusion pump (Ultra 70–3007, Harvard Apparatus Inc., Holliston, MA) under stirring (700 rpm) until completion. A large excess of calcium ions (Ca^2+^ = 0.05 mol) was supplied to facilitate their deployment toward the droplet interface, possibly facilitated by the presence of negatively charged PAA.

Step 5: An aqueous suspension (30 mL; 0.5 M) of sodium carbonate (Na_2_CO_3_) was introduced into the stabilized emulsion at a controlled flow rate (100 μL min^–1^) using an infusion pump until completion. A seeded CaCO_3_ coating was interfacially formed via crystal ripening facilitated by the presence of PAA,^24^ yielding a primary SiO_2_–CaCO_3_–PAA shell. The pH was manually adjusted to 9 using NaOH_aq_ to favor the chemical precipitation of CaCO_3_.

**Figure 1:**
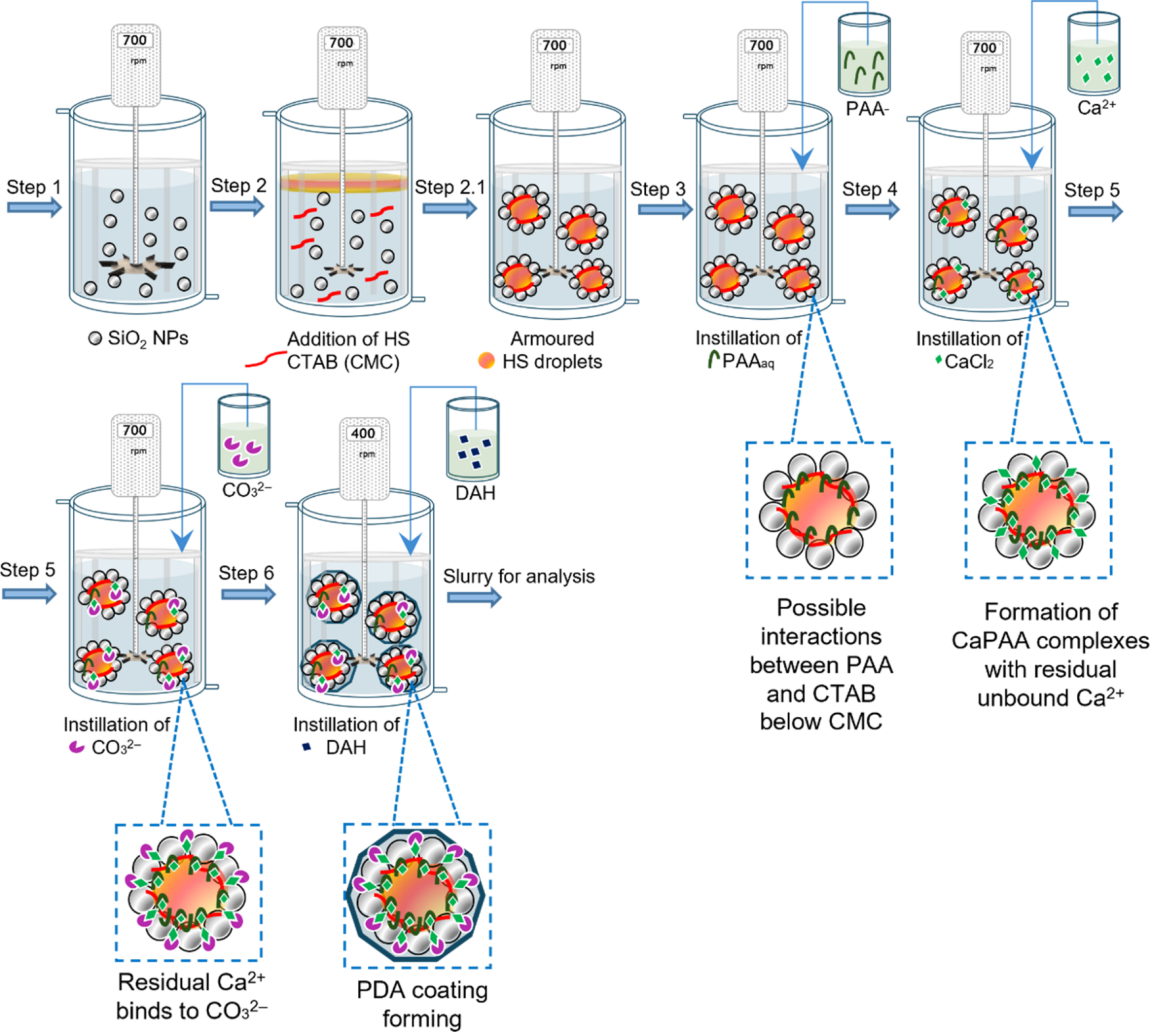
Microencapsulation process of HS via Pickering emulsification into SiO_2_-armored droplets (Step 2, 2.1) in the presence of CTAB at its critical micelle concentration (CMC), followed by sequential instillation of PAA_aq_ (Step 3), Ca^2+^ (Step 4), CO_3_^2–^ (Step 5), and dopamine hydrochloride (DAH; Step 6) to create a multicomponent shell on the surface of the emulsion droplets.

Step 6: An additional coating of polydopamine (PDA) was formed via oxidative autopolymerization (pH 8.5) to promote the structural stability of the CaCO_3_ crystals. A sodium tetraborate tablet was introduced into the microcapsule suspension to create a buffer solution (pH 9.2), to which dopamine hydrochloride (400 mg) was slowly added. The pH was adjusted to 8.5 using HCl_aq_ according to our previous study.^18^ The resulting medium was continuously stirred (400 rpm) over 24 h to enable the complete autopolymerization of dopamine and deposition onto the primary SiO_2_–PAA-CaCO_3_ template, yielding a composite shell wrapped with PDA (SiO_2_–PAA-CaCO_3_–PDA). All solutions (Step 1–6) were thermostated at 25 ± 0.1 °C using a programmable water circulator (Dyneo DD-300F, Julabo Ltd., Stamford, U.K.). Figure 1 represents the key steps of the microencapsulation process.

### 2.3 Fabrication of Microcapsules with Poly(calcium acrylate) Shells

Poly(calcium acrylate) shells were synthesized based on the process described above (Step 1–3). Step 4 was slightly modified to enable the quantitative binding between partially/wholly deprotonated PAA moieties and Ca^2+^ ions. Specifically, two acrylate monomeric units (each with a valency of negative one) react with one Ca^2+^ ion. Given that 1 g of CaCl_2_ (molecular weight (*M*_W_) ≃ 111 g/mol) corresponds to 0.009 mol, it necessitates 0.018 mol of acrylate monomers (MW ≃ 72 g/mol) per gram of calcium chloride for stoichiometric binding. Considering that the aqueous PAA solution (PAA_aq_) is 25% (w/w), 1.0 g of this solution contains 0.25 g of the PAA polymer (p*K*_a_ ∼ 4.5^26,27^), resulting in 3.4 mmol of monomer per gram of solution. Consequently, 5.3 g of the acrylic acid solution is required per gram of calcium chloride to achieve charge neutrality. The pH was adjusted to 8.0 leading to a degree of dissociation of PAA greater than 97%.^26,28^ Accordingly, PAA exhibited a highly negative electrokinetic potential (EKP < −40 mV) due to its ionization into polyacrylate (−CH_2_–CH(−COOH)−)_*n*_ → (−CH_2_–CH(−COO^–^)−)_*n*_ + *n*H^+^,^29,30^ thereby maximizing its binding effectiveness to Ca^2+^ (Supporting Information, Figure S1).

### 2.4 Analytical Techniques

#### 2.4.1 Bright-Field Optical and Fluorescence Sensing Microscopy

Micrographs of microcapsules were captured using a bright field optical microscope equipped with a Motic Pro 252 digital camera (Motic Deutschland GmbH, Wetzlar, Germany, EU) and processed using Motic Images Plus 2.0 software (Motic Deutschland GmbH, Germany, EU). Imaging was performed with PL Fluotar 5×/0.12 and 10×/0.30 objective lenses. To detect fluorescence (from NR-dyed HS), a blue illumination source emitting at 460 nm was utilized in conjunction with a cool-LED pE-300 fluorescence filter. A H3 filter block cube (BP420–490) with a suppression filter (LP 515) and a dichromatic light-splitting mirror (510) were employed to achieve optimal image resolution and field planarity.

#### 2.4.2 Scanning, Cryogenic Scanning, and Transmission Electron Microscopy

Scanning electron microscopy (SEM) was utilized to examine the morphology and surface topography of microcapsules using a benchtop SEM microscope (Hitachi TM3030, Hitachi HighTech, Japan) with a magnification range of 15–30,000X. The instrument was equipped with high resistivity Energy-dispersive X-ray (EDX) silicon drift detectors (SDD) for elemental microanalysis (Bruker Quantax 70, Bruker Corporation, Billerica, MA). The microscope was operated in high vacuum mode (<10^–3^ Pa) with an accelerating voltage of 15 kV. Images were captured using backscattered electron (BSE) detection mode, facilitated by an integrated four-segment detector. Approximately 100 μL of the microcapsule suspension (0.25 wt %) was deposited onto an aluminum pin stub with double-sided adhesive carbon tape. The sample underwent platinum sputtering under vacuum (<10^–2^ Pa) using a Polaron Sputter Coater SC7640 (QuorumTech, Sussex, U.K.) to form an ∼6 nm thick conductive layer prior to SEM imaging.

Cryogenic scanning electron microscopy (CSEM) samples were prepared using a freezing rivet, which was slushed into liquid nitrogen for rapid precooling. The samples were then transferred under vacuum into a Quorum PP3010 cryo-preparation chamber. The sample was freeze-fractured using a cooled knife (−150 °C). An iridium coating was sputtered onto the samples, before being transferred into a Helios G4 CX Dual Beam system (Thermo Fisher Scientific) operating at an accelerating voltage of 2 kV. Cryo conditions (stage: – 140 °C; 2 × 10^–5^ Pa; anticontamination trap cooled to −175 °C) were maintained throughout analysis. The CSEM focused ionized beam (FIB) was operated at a working distance of 5.89 mm, beam current of 100 pA, and energy of 2 keV.

Transmission electron microscopy (TEM) analysis was conducted using a JEOL JEM-2100 (JEOL Ltd., Akishima, Tokyo, Japan) operating at 200 kV. For sample preparation, a small quantity of the capsule sample was combined with LR White resin in a 1.5 mL-Eppendorf tube and left to cure overnight at 60 °C. Once the resin had set, the samples were sectioned using a rotary Biocut ultramicrotome (model 1130, Heildelberg, Germany) into slices being approximately 100 nm thick. These sections were then placed onto specially designed carbon/Formvar-coated TEM copper grids.

#### 2.4.3 Particle Sizing

Particle sizing of microcapsules was performed using laser diffraction with a He–Ne laser beam detector, with a particle detection range of 50 nm to 1 mm (Mastersizer 2000, Malvern Instruments Ltd., Malvern, U.K.). An aliquot (∼5 mL) of microcapsule suspension was added to the continuously stirred dispersing unit (2,000 rpm) preloaded with deionized water (∼120 mL). The analysis was carried out based on the refractive indices (RI_CaCO_3__ ∼ 1.59; RI_PDA_ ∼ 1.49; RI_water_ ∼ 1.33). The Sauter diameter (D_[3,2]_) was determined as the average of five consecutive measurements.

#### 2.4.4 Encapsulation Efficiency and Payload

Both encapsulation efficiency and payload of the microcapsules were determined via UV–vis spectrophotometry, employing a single-standard method, with 36% (v/v) propanol (PrOH_aq_) as the blank.^1^ A small sample of microcapsule slurry (50 mg) was suspended in 20 mL of 36% (v/v) PrOH_aq_ within airtight vials and subjected to ultrasonication over 2 h (VWR International Ltd., USC100TH, Lutterworth, U.K.). The receptor medium was acidified to approximately pH 3 using HCl_aq_. Acidification facilitated the dissolution of the CaCO_3_ in the microcapsules, while ultrasonication ensured the complete rupture of the microcapsule shells, thereby releasing the encapsulated HS into the receptor medium (solubility of HS in 36% (v/v) PrOH > 15 kg·m^–3^).^18^ Subsequent centrifugation (2,000g; 5 min) separated out any insoluble polymeric debris. The supernatant (1 mL) was then analyzed via UV–vis spectrophotometry (CE 2021, Cecil Instruments Ltd., U.K.) operated at the maximum absorption wavelength of HS (306 nm).

#### 2.4.5 Release Studies

The investigation of oil leakage from microcapsules was conducted following the methodology established in our previous work.^31^ Specifically, 20 mg of microcapsule slurry was placed into a dialysis membrane precut to a length of 5 cm (wet diameter 21.3 mm, volume capacity 0.36 mL·mm^–1^, molecular weight cutoff 14 kDa, BioDesign Dialysis Tubing, D004, BioDesign Inc., New York, NY). The membrane underwent pretreatment to eliminate glycerol and other contaminants (sulfides, heavy metals, etc.) potentially present on its surfaces. This involved its immersion in an aqueous solution of EDTA (12 mM) and NaHCO_3_ (2.5% (w/w)), followed by boiling over 15 min. The membrane was then flushed with tap water to remove glycerol, and subsequently rinsed thoroughly with demineralized water. Each membrane was loaded with 2 mL of PrOH_aq_ at pH 9, folded twice, and securely pegged at both ends. The membrane was submerged in the receptor medium (100 mL PrOH_aq_ pH 9) and subjected to magnetic stirring at room temperature (22 ± 1 °C). Oil leakage was tracked over time by withdrawing aliquots (1 mL) from the receptor medium and measured via UV–vis spectrophotometry (306 nm), utilizing a quartz cuvette with a 10 mm path length (Hellma Analytics UK Ltd., Essex, U.K.). To maintain sink conditions, each aliquot withdrawal was immediately followed by the addition of 1 mL of fresh PrOH_aq_ to the receptor medium. It is worth mentioning that aqueous propanol was selected as a receptor medium instead of water because the solubility of the core oil in it is significantly higher than in water, which can be used as an accelerated test to determine the diffusivity of the core oil in the shell of microcapsules when exposed to aqueous environment. Moreover, in some formulations, such as those in cosmetics, pharmaceuticals, or food products, which often contain alcohols or other organic components, direct measurement of the oil release from microcapsules to aqueous propanol is directly relevant to their industrial applications.

#### 2.4.6 Mechanical Properties Determined Using Micromanipulation

The mechanical properties of individual microcapsules were evaluated using a parallel plate compression micromanipulation technique.^32^ This was performed with a custom-built apparatus, which included a side-view high-speed camera (4912–5010/000, Cohu, CA).^33^ A finely polished flat-end glass tube (∼50 μm) as the output attachment was connected to a force transducer (series: 405A, model:405010; executable sensitivity of 0.9532 mN·V^–1^). Approximately 0.15 mL of a diluted microcapsule suspension was dispensed in two droplets onto a precut glass slide (∼2.5 cm^2^, ∼1 mm thickness). The slide was then securely mounted onto the rig stage. Thirty individual microcapsules were compressed at a speed of 10.0 μm·s^–1^. The mechanical response generated was analyzed to determine the force versus displacement for each microcapsule. System compliance was calibrated thrice to allow measurement of the distance traveled by the force probe (displacement) during the compression of each microcapsule to rupture.^34^

#### 2.4.7 Fourier Transform Infrared Spectroscopy (FT-IR)

Fourier-transform infrared (FT-IR) spectra of both solid and liquid samples were recorded using a PerkinElmer FT-IR GX System, equipped with an attenuated total reflection (ATR) accessory (DuraSampleIR). Each spectrum was averaged over 200 scans, with the wavenumber spanning 4,000 to 400 cm^–1^, with a resolution of 4.0 cm^–1^. Solid samples (∼8 mg) were homogeneously mixed within an anhydrous KBr matrix (0.5 g), acting as an IR-transparent medium, and compressed into pellets using a semiautomatic rotary tablet press (LFA Tablet Presses, U.K.) at 80 kN for 120 s. The sample-to-KBr weight ratio of 0.015 was chosen to prevent the formation of opaque pellets, ensuring adequate IR beam transmission and optimal spectral quality.^1^ To obtain free-flowing powder samples of microcapsules suitable for FT-IR analysis, approximately 15 mL of the microcapsule suspension was placed in prepierced polyethylene grip seal bags (5 × 7 cm^2^) and frozen at −21 °C for 8 h. The freshly frozen microcapsules were then freeze-dried using a ScanVac CoolSafe Pro (DVS1000, Labogen, Lillerød, Denmark, EU) under vacuum conditions (0.04 kPa) at −53 °C until completely dry.

#### 2.4.8 Flame Atomic Absorption Spectroscopy (AAS)

The residual Ca^2+^ concentrations (unreacted metal cations) after cross-linking of PAA were quantitatively determined using atomic absorption spectroscopy (AAS; Agilent 240FS, Agilent Technologies, Palo Alto, CA) using a wavelength of 423 nm. Each sample (7 in total) was prepared with 1.0 g of PAA (25 wt %) in water (50 mL), at increasing concentrations of CaCl_2_ (0.4, 1.0, 2.0, 3.0, 4.0, 5.0, 8.0 g/L), according to Section 2.3. Following Ca-PAA binding, all samples were then centrifuged at 2000*g* for 10 min (Megafuge 16R, Thermo Scientific) to remove Ca-PAA gels. The supernatant was then passed through a 0.2 μm syringe filter prior to analysis. Calibration standards at specific concentrations were prepared to produce a calibration curve (0–200 ppm). Quality control (QC) was maintained by running calibration checks after every 5 samples, with recalibration performed if drift exceeded a 5% error margin.

#### 2.4.9 Statistical Analysis

Unless otherwise noted, each set of experiments was conducted in triplicate. Variability among the results is presented as the mean value ± standard error (St. E).

## 3 Results and Discussion

### 3.1 SiO_2_–PAA-CaCO_3_ Microcapsules

**Figure 2:**
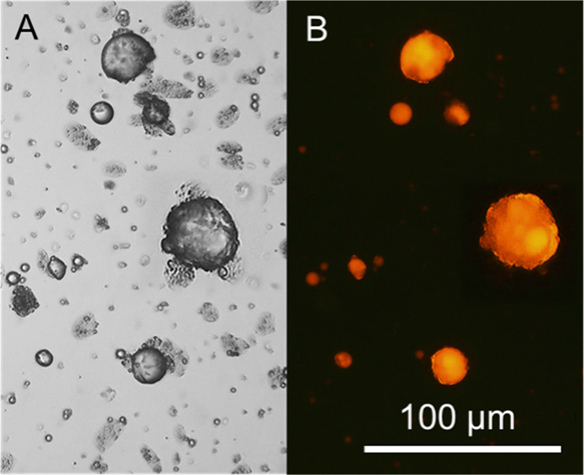
Optical micrographs of suspended primary SiO_2_–PAA-CaCO_3_ microcapsules observed under (A) bright-field applying an automatic white balance compensation filter (B) and fluorescent blue light (operational spectral wavelength ∼460 nm).

Figure 2 illustrates polydisperse primary microcapsules, dispersed in water, with a shell composed of SiO_2_–PAA-CaCO_3_. Bright-field topographical analysis revealed a continuous and rather smooth shell, with a relatively spherical morphology (Figure 2A). However, irregular invaginations at their peripheries were also observed, yielding localized wrinkling on the capsule surfaces and discontinuities in their spherical outlines. Similar structural features, including deviation from sphericity and invaginations, have been reported in other studies on PAA-based structures, such as irregularly shaped, partially porous PAA-cysteine microparticles.^35^

The peripheral indentations of the microcapsules may be attributed to the hydrophilic nature of PAA, which may form highly swellable hydrogel-based shells.^36^ Superporous poly(acrylic acid) hydrogel microparticles have been documented by Yang et al.,^36^ featuring a unique porous structure that occurs in an amorphous state, capable of swelling up to 80 times (volume/weight of a dry sample) in distilled water.

Furthermore, the potential presence of electrostatic interactions between opposing charged groups forming the shell cannot be precluded. Such interactions could lead to irregular rearrangements/stacking of the NPs at the interface and through the shell thickness.^31^

**Figure 3:**
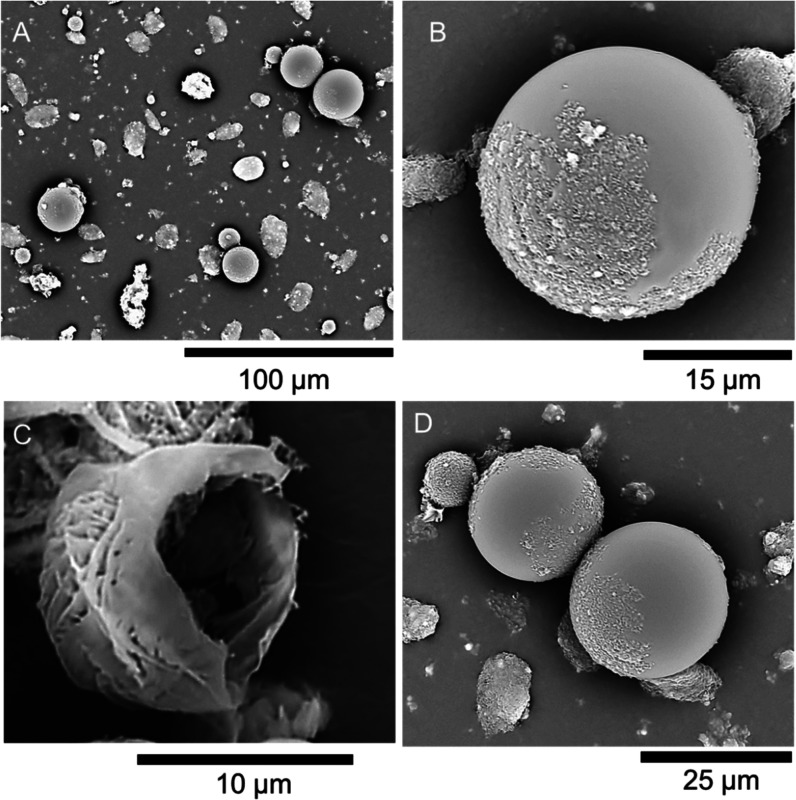
SEM micrographs of primary SiO_2_–PAA-CaCO_3_ microcapsules: (A) overview of microcapsules; (B) close-up of an individual microcapsule, exhibiting a spherical morphology, with both smooth and rough surface areas; (C) incomplete microcapsule with a smooth-coarse surface, displaying a core-thick shell structure; (D) close-up of two individual spherical microcapsules, both featuring a smooth-coarse surface.

Under fluorescence, a consistently strong red-emitting signal was detected, indicating the effective encapsulation of the dye-stained active beneath the coatings. In addition, the microcapsule surface appeared relatively compact, without any evident cracks (Figure 2B). Huang et al.^37^ reported that coordinated structures between polyanionic PAA^–^ and Ca^2+^ ions are induced through their complexation with H_2_O molecules.^37^ In their work, controlling the kinetics of interaction between the two species enabled potential regulation of CaCO_3_ mineralization. At pH 9, the controlled instillation of carbonate ions during a PAA–Ca^2+^ complexation process yielded consistently shaped colloidal particles. This principle may apply to our system, with the distinction that the colloidal particles are deposited onto the NP-armored interface in the presence of PAA, leading to distinct morphologies. Thus, the relatively spherical PAA-CaCO_3_ shells may not form directly from the arrangement of colloidal matter around the armored shells, but rather as a result of the precipitation rate of the binding species (PAA, Ca^2+^, CO_3_^2–^) and the MW of PAA during complexation, as well as the fluid mixing within the vessel. As noted by Huang et al., a more disordered (entropic) coordination structure of the PAA–Ca^2+^–H_2_O complexes may form with shorter complexation time, which can dramatically affect the structural and mechanical stability of the resulting complexes. Accordingly, in our system, reacting ions were instilled at a low, controlled, flow rate (100–200 μL/min), leading to the formation of relatively spherical microcapsules, as demonstrated in the subsequent SEM analysis shown in Figure 3.

#### 3.1.1 Surface Topography and Structural Analysis of SiO_2_–PAA-CaCO_3_ Microcapsules

Figure 3A displays microcapsules with a predominantly spherical morphology. The microcapsules exhibited rather similar sizes, in the range of 20–25 μm. The outer surface of microcapsules appeared relatively smooth and wrinkle-free, despite the presence of several irregularly shaped crusts. These crusts were likely due to the presence of excess material that did not complex onto the surface, possibly adhering at a later stage (e.g., during drying for the SEM preparation). Flattened structures were also present, which looked like precipitates resulting from the polymer-ions (PAA-Ca^2+^) interactions.

Figure 3B provides a close-up of a complete SiO_2_–PAA-CaCO_3_ microcapsule with an inhomogeneous surface. As can be seen, approximately half of the exposed surface appeared consistently smooth, probably due to the continuous development of smooth polymeric PAA-dominated complexes around the armored core. In contrast, the other half exhibited a coarse texture, with dimples and surface defects.

The microcapsules likely possessed a mesoporous structure, characterized by a dense microporous mantle of CaCO_3_ crystals interspersed with PAA-CaCO_3_ complexes. Additionally, a smaller porous lump was visible, which could result from mineralization of CaCO_3_ crystals in bulk, deposited onto the relatively tough/soft surface during/after encapsulation. The resulting crystals are likely calcite, the most thermodynamically stable polymorph, as our process is thermostated at 25 °C.^38^ At this temperature, calcite predominantly forms on soft and hard surfaces, whereas other polymorphs of CaCO_3_, such as vaterite (∼40 °C) and aragonite (∼55–70 °C), are less thermodynamically stable and may commonly be observed at higher temperatures.^38,39^ Alternatively, these uneven lumps might have been triggered by the excess inorganic NPs spontaneously aggregating, complexing, or depositing nonuniformly at the interface during encapsulation.

EDX is well suited for bulk elemental composition, with a micron-scale penetration depth (∼0.5–2 μm), depending on the accelerating voltage of the electron beam.^40^ It revealed that the smooth surface texture of the microcapsules contained approximately 88–92% atomic carbon, and around 1–5% atomic calcium (Supporting Information, Figure S2 and Table S2). Interestingly, the coarse texture showed a similar detection range for atomic carbon (∼86%), along with over 1% elemental silicon and 9% oxygen (Supporting Information, Figure S3 and Table S3). Although not fully conclusive, due to the limitations of the EDX penetration depth, these findings suggest the presence of excess SiO_2_ NPs irregularly deployed on the shell. Furthermore, it cannot be ruled out that SiO_2_ NPs may aggregate prior to adsorbing onto the surface of the emulsion droplets. The small beads present on the surface exhibited a consistently similar composition, although their internal structure remains unknown, and the possibility of oil pockets within them cannot be excluded.

Incomplete microcapsules were also observed, particularly those with unsealed shells (Figure 3C). These observations provided insights into both the morphological and structural configurations of the microcapsules. The presence of an oil-hosting pocket within the microcapsules was evident, suggesting a core–shell structure with a relatively thick shell. The surface of the microcapsules appeared relatively smooth, despite the presence of randomly orientated ripples, possibly resulting from the formation of irregular Ca-PAA complexes.

A close-up of two individual microcapsules is presented in Figure 3D. Large portions of the microcapsule surfaces appear smooth, indicating uniform solidification of the shell. However, roughened areas with granular textures are visible on parts of the surfaces, particularly around the upper hemisphere of the left particle and the lower hemisphere of the right particle (Figure 3D).

### 3.2 Surface Topography of SiO_2_–PAA-CaCO_3_–PDA Microcapsules

**Figure 4:**
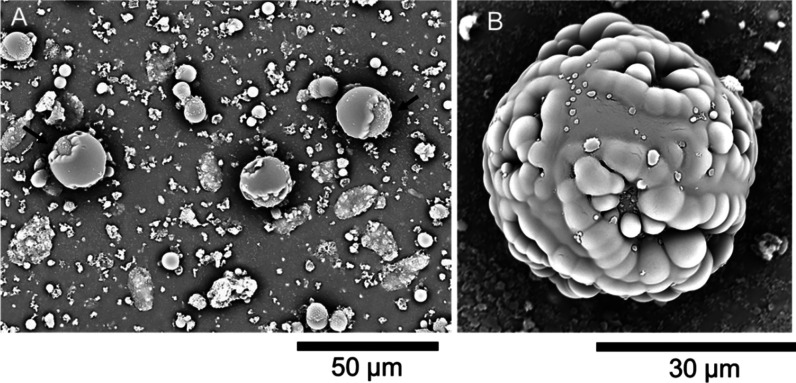
SEM micrographs of composite microcapsules with an outermost coating of PDA: (A) Overview of microcapsules partially wrapped with PDA (black arrows indicate the primary microcapsules as the inner template); (B) close-up of an individual microcapsule, exhibiting a spherical morphology, and almost fully wrapped with PDA (accelerating voltage 30 kV).

Figure 4A displays SEM micrographs of composite microcapsules being wrapped with PDA. Specifically, a group of self-isolated microcapsules is visible, each encased in a soft PDA wrapping around the primary microcapsule templates (see black arrows). There appeared to be a moderately thick PDA-based shell, which exhibited a smoother surface compared to that of primary microcapsules. A close-up topographical examination of an individual microcapsule (diameter ∼35 μm) revealed a highly textured, spheroidal microcapsule with a complex surface morphology (Figure 4B). The PDA wrapping forms a pseudocontinuous layer after ∼24 h, characterized by an amorphous structure that adheres onto the primary surface in a uniform manner. This layer comprised multiple rounded, bulbous interconnected protrusions, giving the overall appearance a bumpy or lobate-like texture.

The presence of rounded lobes might be indicative of specific growth patterns, which are compatible with the alkaline-autopolymerization of PDA, likely resulting in continuous, smooth yet bumpy films, as also reported in other works.^41^ Additionally, small, scattered nodules or granules are observed across the outermost surface (Figure 4B), which may represent secondary deposits, such as excess material, adhering to the primary structure possibly during the autopolymerization of dopamine.

Subsequent EDX analysis revealed that the elemental composition of the outermost coating consists of approximately 91–93% elemental carbon, which aligns with the expected composition of the PDA wrapping (Supporting Information, Figure S4 and Table S4), and nearly 1–2% calcium. In contrast, EDX analysis of the inner template (primary microcapsule) detected over 1% silicon, likely originating from the SiO_2_ nanoparticles. Additionally, a significantly higher calcium content (7–8%) was observed, along with approximately 88% carbon, possibly indicating the presence of Ca-PAA complexes. The presence of Ca-PAA was further confirmed by FTIR spectroscopy, as discussed in Section 3.5.

#### 3.2.1 Structural Analysis of SiO_2_–PAA-CaCO_3_–PDA Microcapsules

**Figure 5:**
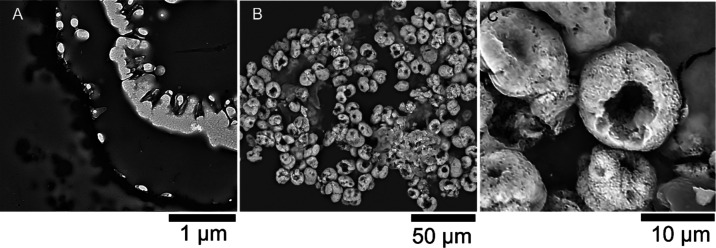
(A) Close-up TEM micrograph of a composite microcapsule cross-section, illustrating both inorganic (innermost, brighter phase) and organic (detached, darker phase) coatings; (B) SEM micrograph providing an overview of microcapsules postrelease in aqueous propanol; (C) close-up SEM micrograph of an incomplete thick-shell microcapsule: an aliquot of alcohol-rich suspension containing the “spent microcapsules” (i.e., post release) is withdrawn from dialysis and left to dry on SEM stub.

Figure 5A displays a detailed TEM micrograph of a portion of a microcapsule cross-section. The contrast in the TEM image indicates materials with varying electron densities, suggesting the presence of a heterogeneous/multicomposite structure (detached CaCO_3_–PAA layers). Figure 5B presents a cluster of small, rounded, doughnut-like microcapsules densely packed together. These microcapsules were collected to qualitatively assess their structural integrity after release in aqueous propanol. They appeared relatively uniform in shape, with diameters ranging approximately from 5 to 10 μm. Their featured central depressions or holes indicated a hollow structure (Figure 5B), which also enabled the estimation of their shell thickness (approximate range 2.4–3.0 μm). However, since the microcapsules only displayed front-facing holes, the possibility that this damage was caused by direct electron radiation cannot be ruled out (Figure 5C). Alternatively, this may have been caused by solvent-induced stress or chemical perturbation due to prolonged exposure to aqueous propanol. No visible polydopamine (PDA) coating was observed postrelease. This suggests that PDA may be structurally weakened and partially/fully dissolved when exposed to hydroalcoholic media over extended periods.^42^

Additional characterization result is provided in Supporting Information, Figure S5, which shows a cryo-SEM micrograph of a microcapsule, in its hydrated state at cryogenic temperatures, before and after rapid FIB slicing. The corresponding EDX analysis confirmed the presence of three predominant elements in a multilayer structure (see Supporting Information, Figure S6).

### 3.3 Microcapsules with Poly(calcium acrylate) Shells

**Figure 6:**
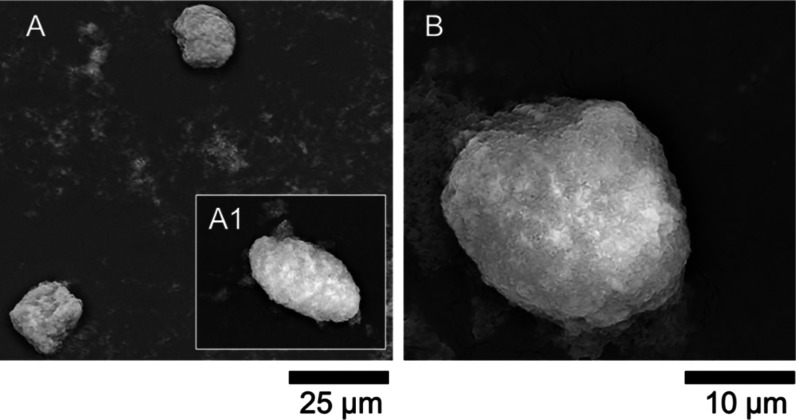
SEM micrographs of (A) blank microcapsules with a poly(calcium acrylate) shell, fabricated without carbonate (CO_3_^2–^) ions, with a relatively spherical and (A1) elongated morphology, and (B) close-up view of a relatively spherical microcapsule.

Figure 6 displays two SEM micrographs of microcapsules featuring a poly(calcium acrylate) shell (Ca-PAA), fabricated in the absence of CO_3_^2–^. The particles appeared rather irregular in shape, with a possibly granular surface (Figure 6A). The inset (A1) highlights a clear view of a microcapsule with an ellipsoidal morphology. Nonspherical morphologies are often reported in the literature, as also documented in our previous works for different chemistries.^1,43^ The close-up shown in Figure 6B depicts a particle which appears to be spherical or slightly oval in shape, with a rough, textured surface. This may suggest a complex, possibly crystalline or agglomerated structure, likely due to the binding between Ca^2+^ and PAA, as well as the inclusion of SiO_2_ NPs across the structure. FTIR analysis is presented in Section 3.5. Additional evidence is provided in Supporting Information, Figure S7. Optical microscopy revealed that Ca-PAA microcapsules tended to aggregate into clusters. Their shell structures appeared to merge into complexes with irregular morphologies (indicated by red arrows and insets A, B), although individual hydrophobic cores remained visible. While the formation of armored hydrophobic cores via Pickering stabilization was advantageous in preventing coalescence, the Ca-PAA shells may exhibit stickiness, leading to agglomeration near their isoelectric point (electroneutralisation).

### 3.4 FTIR Analysis

**Figure 7:**
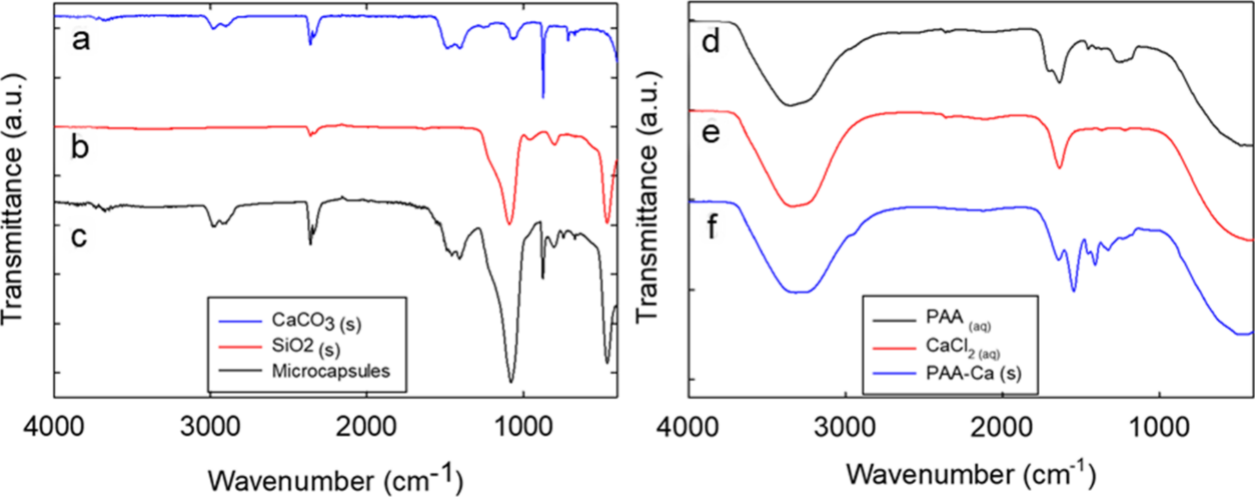
FT-IR spectra of solid (a) CaCO_3_, (b) SiO_2_, (c) composite (PAA-SiO_2_–CaCO_3_) microcapsules, (d) aqueous PAA, (e) CaCl_2_, (f) wet PAA-Ca complexes.

All relevant solid materials used in the microencapsulation were assayed for their FTIR spectra. On the left-hand side of Figure 7, the spectra of (a) commercial CaCO_3_, (b) SiO_2_, (c) composite (PAA-SiO_2_–CaCO_3_) microcapsules are presented. As can be seen, c exhibited distinct absorption bands corresponding to specific molecular vibrations that tracked similarities with a and b. This suggests the coexistence of different entangled layers within the composite shell, being silica and CaCO_3_.

Specifically, the spectrum (a) presents a relatively strong, broad absorption band (∼1400 cm^–1^) corresponding to the asymmetric stretching vibration (ν_3_) of the carbonate ion (CO_3_^2–^). Additional medium-intensity bands were detected at ∼872 cm^–1^ and ∼712 cm^–1^, which were possibly attributed to the out-of-plane and in-plane bending (ν_2_) of the carbonate ions. These suggest the characteristic absorption bands of calcite^44^ at 877 and 712 cm^–1^, however overlapping with the absorption bands of vaterite is also plausible at ∼877 cm^–1^.^45,46^ No characteristic band typically ascribable to aragonite (∼700–701 cm^–1^) was detected in our experiments.^47,48^

A weak broad band was also obvious at around ∼3000 cm^–1^, which is likely associated with stretching vibrations of moisture (−OH), possibly adsorbed from atmospheric air during FTIR sampling.

The spectrum of SiO_2_ (b) includes its typical absorption bands: a strong, broad band around ∼1086 cm^–1^ corresponding to Si–O–Si asymmetric stretching vibrations, a band near ∼794 cm^–1^ associated with symmetric stretching of Si–O–Si, a weaker band around 462 cm^–1^ likely due to Si–O–Si bending vibrations. An additional band at around 2350 cm^–1^ was detected, which might be attributed to overtone and/or combination bands, or possibly to the CO_2_ gas phase adsorption onto the surface of silica. Similar results were reported by Li et al.^49^

The FTIR spectrum of primary SiO_2_–CaCO_3_–PAA microcapsules (c) reveals a combination of features from both calcium carbonate (CaCO_3_) and silicon dioxide (SiO_2_), along with additional bands likely due to organic heteroinclusions. A band around ∼1440 cm^–1^ suggests the presence of carbonate groups, while an intense band near ∼1085 cm^–1^ indicates Si–O–Si stretching. A relatively wide absorption band in the ∼2900–3000 cm^–1^ range suggests C–H stretching vibrations, possibly due to the presence of other organic compounds (e.g., PAA). Moreover, a tentative broad band around ∼3500 cm^–1^ was also detected, indicating the stretching vibrations of O–H bonds (e.g., moisture).

It is worth mentioning that no FTIR pattern of polydopamine was presented as its characteristic absorption bands are well-known.^50,51^ More importantly, it exhibits absorption bands similar to PAA, potentially creating overtone/band overlapping when deposited onto primary microcapsules (e.g., indolic C–C, C=O (∼1620–1725 cm^–1^), C–O (∼1100–1300 cm^–1^), and N–H and O–H stretching (∼3250–3500 cm^–1^)). Nonetheless, the formation of the polymer was confirmed through visual observations during the experiment; at the required pH 8.5, the microcapsule suspension turned deep dark brown within 24 h, as reported in the literature.^18^

The figure on the right-hand side of Figure 7 presents the spectra of PAA, as well as its interaction with calcium ions (Ca^2+^) in both wet and dry states. When dealing with the aqueous PAA spectrum (d), a broad O–H stretching band around 3250–3500 cm^–1^ and a strong C=O stretching band near 1690 cm^–1^ are observed, characteristic of carboxylic acid groups.

Upon the introduction of Ca^2+^ (solid PAA-Ca in suspension (f)), significant changes occurred, particularly in the C=O stretching region, indicating complexation between PAA and Ca^2+^. These shifts are significantly pronounced, suggesting the effective coordination between carboxylate groups and Ca^2+^ ions. For completion, the spectrum of CaCl_2_ (e) is also provided, dominated by a broad water band around 3400 cm^–1^ and lacking the broader C=O band seen in PAA, yet showing a narrower characteristic peak at ∼1625 cm^–1^.

### 3.5 Binding Mechanism between Ca^2+^ and Acrylic Monomers

Quantification of the binding constant between poly(acrylic acid) (PAA) and calcium ions (Ca^2+^) is a measure of the affinity between the polymer and the metal ion, reflecting the strength of their interaction. Carboxylate groups in PAA can chelate calcium ions above their p*K*_a_, forming a stable complex as shown by the reaction^52^
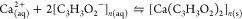
1

**Figure 8:**
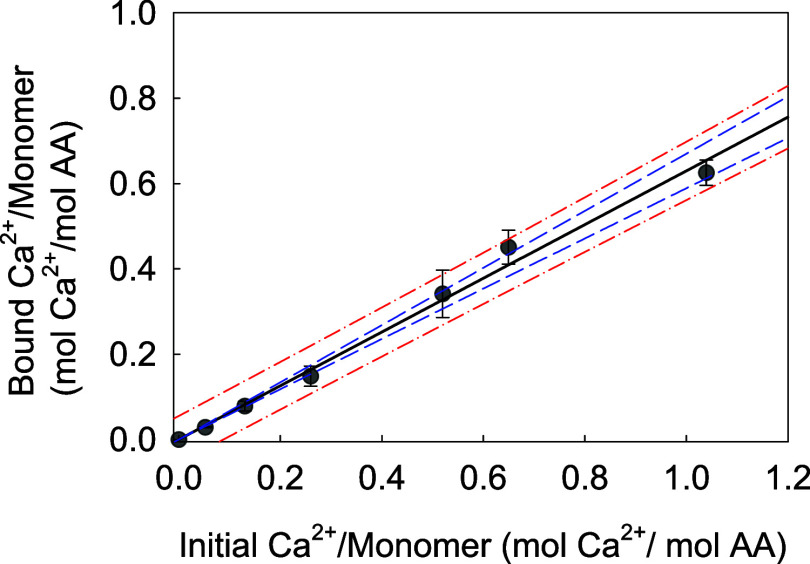
AAS-determined bound Ca^2+^ to monomer molar ratio (*y*) plotted against the initial Ca^2+^ to monomer ratio (*x*); The linear model (*y* = *y*_0_ + *kx*) applied to the data showed *y*_0_ ≈ 0 mg and a proportionality constant *k* = 0.63 ± 0.02 and indicates a proportional rate of consumption of Ca^2+^ binding to PAA; the model exhibited a high degree of fit, as evidenced by the coefficient of determination (*R*^2^ > 0.991) and the statistical significance level (*p* < 0.0001). The curve-fit linear regression was generated via Sigma Plot 15.0 (Alfasoft, Gothenburg, Sweden, EU). Confidence interval of the mean (− −; blue) and predicted (−·–; red) values are also displayed.

Figure 8 displays the relationship between the molar ratio of calcium bound ([Ca]_b_) to monomeric AA units (*n*_AA_*) and the molar ratio of the initial calcium concentration ([Ca]_0_) to the monomeric AA (see Section 2.3). Briefly, [Ca]_b_ was calculated as the difference between the initial moles of dissociated CaCl_2_ and the remaining moles of Ca^2+^ measured by AAS, whereas *n*_AA_* was simply the ratio between the effective mass of PAA (0.25g) and the molecular weight of its monomeric unit (∼72.1 g/mol).

A strong linear trend (coefficient of determination, *R*^2^ > 0.99) was observed, suggesting that the complexation of Ca^2+^ to the monomeric segments occurs in a proportional manner, with the increase of the initial calcium concentration. This likely indicates a first-order binding mechanism, where each calcium ion binds directly to the corresponding sites on the monomer, with identical structure and affinity.

More specifically, as the ratio of Ca^2+^ to AA repeat units increases, the amount of calcium bound per unit also rises, with a rate of increase or binding gradient (BG) around 0.63. Given that the stoichiometric ratio (SR) of AA to Ca^2+^ is approximately 0.5, assuming complete ionization of the AA units at pH 8 (100% monomeric sites activated), this suggests that the degree of cross-linking—and consequently, the gelling between the two species—can be effectively controlled. This likely occurred without cooperative effects or allosteric mechanisms that might otherwise deplete the binding affinity of neighboring subunits.

However, a potential discrepancy (i.e., SR = 0.5 vs BG = 0.63) could lie in the actual concentration of PAA. The supplier of poly(acrylic acid) specifies a range of 23–27 wt %, whereas a midpoint of 25% was assumed. Based on the data, a novel single-pathway binding constant (SPBC = 0.63 ± 0.02), corresponding to the BG slope, can be defined. SPBC is dimensionless and system specific, offering a clear-cut insight into the efficiency and extent of calcium (Ca^2+^) binding per monomer unit (acrylic acid, AA) within the gel formation process, in comparison to conventional stoichiometry.

The data seem to confirm that gelation and the resulting properties of the microcapsules may be significantly influenced by Ca^2+^ cross-linking. Besides, the presence of possible site-to-site competing reactions cannot be ruled out, affecting the BG value.

This tunability implies that the extent of cross-linking (and therefore gelling) can be controlled by simply manipulating the initial Ca^2+^ concentration, which can, in turn, affect the structural continuity, porosity, and mechanical properties of the resulting microcapsules Similar conclusions were drawn by Zhong et al., reporting that the mechanical properties of PAA hydrogels can be highly influenced by the concentration of cross-linkers, water molecules and metal (Fe^3+^) ions.^22^

As described by Donnet et al.,^24^ it is imperative that the monomeric content is optimized since the binding mechanism is significantly inhibited when the PAA concentration is too high, especially for the growth of seeded calcium carbonate.

### 3.6 Particle Size Distribution

**Figure 9:**
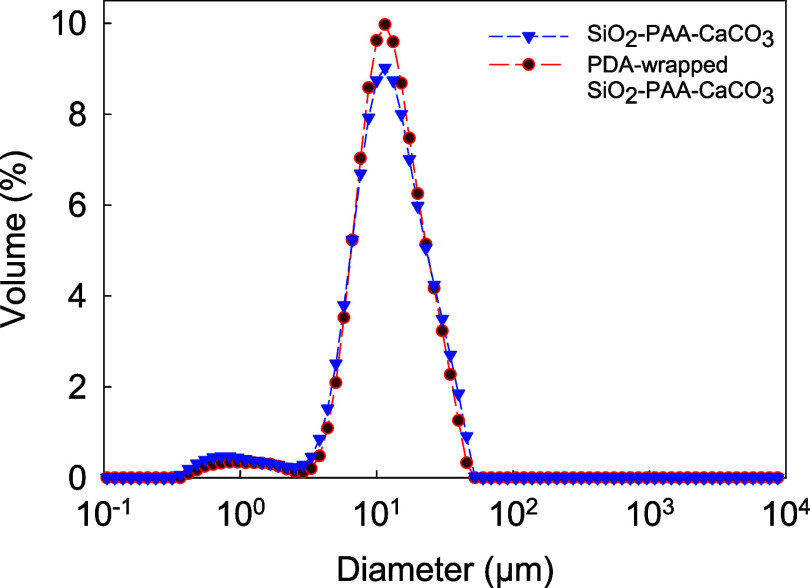
Particle Size Distribution of SiO_2_–PAA-CaCO_3_ (▼) and PDA-wrapped SiO_2_–PAA-CaCO_3_ composite (●) microcapsules.

The particle size distributions (PSD) of primary SiO_2_–PAA-CaCO_3_ and PDA-wrapped SiO_2_–PAA-CaCO_3_ are shown in Figure 9. Specifically, the PSD of primary microcapsules (blue curve) is centered at a rather small diameter (*D*_3,2_ = 5.4 ± 0.1 μm), featuring a relatively narrow and sharp peak, indicating a certain degree of homogeneity. There is a small secondary peak at a smaller size range (∼1 μm), suggesting the presence of a bimodal distribution, possibly due to NP-based aggregates or debris formed in the bulk during encapsulation.

PDA-wrapped SiO_2_–PAA-CaCO_3_ (red curve) microcapsules yielded a single-peak PSD, approximately ranging 3–100 μm, similar to that of primary microcapsules. However, the Sauter diameter was determined to be slightly higher (*D*_3,2_ = 8.8 ± 0.3 μm), which is likely due to the PDA-based wrapping, as shown in Figure 4. Additionally, the peak was slightly broader than that observed for the SiO_2_–PAA-CaCO_3_ capsules, and the curve appeared somewhat asymmetric, suggesting a possible skew in the PSD, owing to the effect of PDA possibly layering inhomogeneously around the primary microcapsules.^18^ The key PSD parameters are listed in Table 1.

**Table 1:** Key Particle Size Distribution Parameters^a^

	*D*_3,2_	*D*_4,3_	*D*_10%_	*D*_50%_	*D*_90%_	SPAN
SiO_2_–PAA-CaCO_3_ (▼)	5.4 ± 0.1	20.7 ± 0.2	4.1 ± 0.1	16.3 ± 0.1	43.1 ± 0.5	2.39
PDA-wrapped composite (●)	8.8 ± 0.3	15.9 ± 0.1	6.5 ± 0.1	13.5 ± 0.1	29.6 ± 0.1	1.70

a *D*_3,2_ represents the surface-weighted Sauter mean diameter, which gives an average particle size based on surface area; *D*_4,3_ is the volume-weighted De Brouckere mean diameter, reflecting the average particle size based on volume; the percentiles *D*_10%_, *D*_50%_ (median), and *D*_90%_ indicate the particle diameters below which 10, 50, and 90% of the sample’s volume is found, respectively. All the aforementioned diameter (*D*) values are expressed in μm. SPAN of a particle size distribution is a dimensionless measure of the width of the distribution and is an indicator of the uniformity of the particle sizes [SPAN = (*D*_90%_ – *D*_10%_)/*D*_50%_].

### 3.7 Barrier Properties

**Figure 10:**
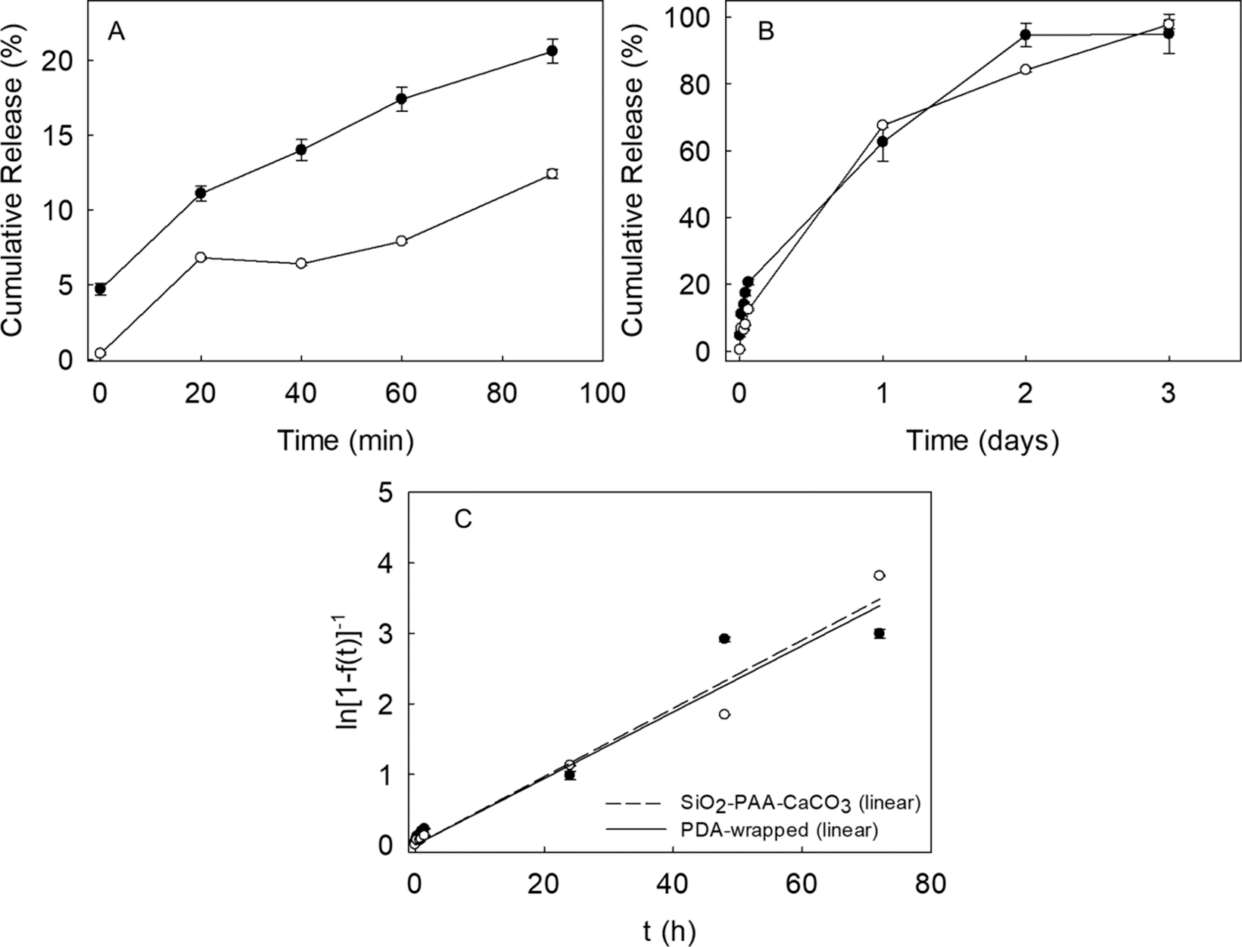
(A) Short-term (<2 h) and (B) long-term (∼3 days) cumulative release of HS from primary SiO_2_–PAA-CaCO_3_ (**●**) and PDA-wrapped SiO_2_–PAA-CaCO_3_ composite (o) microcapsules in aqueous propanol (36% v/v); Point-to-point segments only represent the trend of the data points. (C) Curve-fit nonlinear regressions [*f*(*t*)=1 – exp(−*t*/τ)] for the cumulative release (experimental data), linearized into ln[1 – *f*(*t*)]^−1^ = (1/τ)*t*, and processed via Sigma Plot 15.0 (*p* < 0.0001). Some error bars are smaller than the size of symbols and thus not visible.

Figure 10 presents the release profiles of HS from both primary SiO_2_–PAA-CaCO_3_ (encapsulation efficiency 58.6 ± 1.8%; payload 16.6 ± 0.5%) and PDA-wrapped SiO_2_–PAA-CaCO_3_ (encapsulation efficiency 67.1 ± 2.7%; payload 19.8 ± 0.8%) microcapsules, obtained from different batches, over short (A) and more extended (B) time periods, in aqueous propanol. The encapsulation efficiency and payload values between primary and PDA-wrapped SiO_2_–PAA-CaCO_3_ were not statistically different. It is important to note that the solubility of HS in 36% (v/v) aqueous propanol (∼5 kg/m^3^ at 25 °C^53^) is almost 3 orders of magnitude greater than that in water (∼10 ^–2^ kg/m^3^),^18,54^ which can significantly increase the concentration gradient (driving force), thus accelerating the release kinetics.

Figure 10A covers a period of less than 2 h, suggesting that the primary microcapsules exhibited a steady release profile, reaching approximately 20% after ∼80 min. In contrast, the PDA-wrapped microcapsules showed a delayed release, reaching just above 10% after ∼80 min. This suggests that the PDA coating (Figure 4B) introduced an additional mass transfer resistance, effectively reducing the HS leakage rate into the aqueous propanolic medium.

Figure 10B illustrates the long-term release behavior, showing that nearly 100% release (corresponding to the whole oil load cumulatively present in the capsules) was achieved by both microcapsule types after 3 days. Given the high solubility of HS in aqueous propanol, this result was not surprising. The release profiles of both microcapsule types were similar over this extended period, indicating that the PDA wrapping primarily influenced only the short-term release. Prolonged exposure of the PDA coating to the hydro-alcoholic medium may compromise its structural integrity, potentially leading to its partial or complete solubilization. This seemed consistent with the postrelease SEM micrographs (Figure 5C,D).

Figure 10C depicts curve-fit nonlinear regressions [*f*(*t*) = 1 – exp(−*t*/τ)] for the experimental cumulative release from microcapsules. When linearized into ln[1 – *f*(*t*)]^−1^ = (1/τ)*t*, it enables the determination of the characteristic diffusion time (τ) of HS through the capsule shell in aqueous propanol, corresponding to a period of approximately 3 days. Both data sets exhibited a strong linear relationship, indicative of a specific kinetic release rate, possibly first-order, driven by the high concentration gradient in 36% (v/v) propanol. The linear regressions aligned closely at short times, and the corresponding characteristic diffusion times were found to be 20.7 ± 1.1 h (coefficient of determination *R*^2^**≈** 0.97; *p* < 0.0001) and 21.2 ± 1.6 h (*R*^2^**≈** 0.94; *p* < 0.0001) for the SiO_2_–PAA-CaCO_3_ and PDA-wrapped microcapsules, respectively. Although the linear trends tentatively diverged at longer times, there was no statistically significant difference between the two τ values. This suggests that the PDA wrapping may dissolve upon prolonged exposure to hydro-propanol media. Consequently, at longer times, the resistance to mass transfer is only governed by the composite SiO_2_–PAA-CaCO_3_ shell. Nonetheless, the PDA wrapping may still provide an additional mass transfer resistance during the initial stages (<2 h).

The characteristic diffusion times seemed to be higher than that previously reported by Bhutkar et al.^53^ (10–13 days), under similar conditions (hexyl salicylate into aqueous propanol). They investigated the barrier properties of novel microplastic-free microcapsules fabricated by bis-urea self-assembly.

These findings suggest that these new microcapsules could be suitable for applications requiring controlled and sustained release, with the full load delivered within 3 days. It is important to note that release in purely aqueous environments is expected to be dramatically slower, potentially extending over several months, due to the lower concentration driving force, as discussed in our previous studies.^18,31^

### 3.8 Mechanical Properties

**Figure 11:**
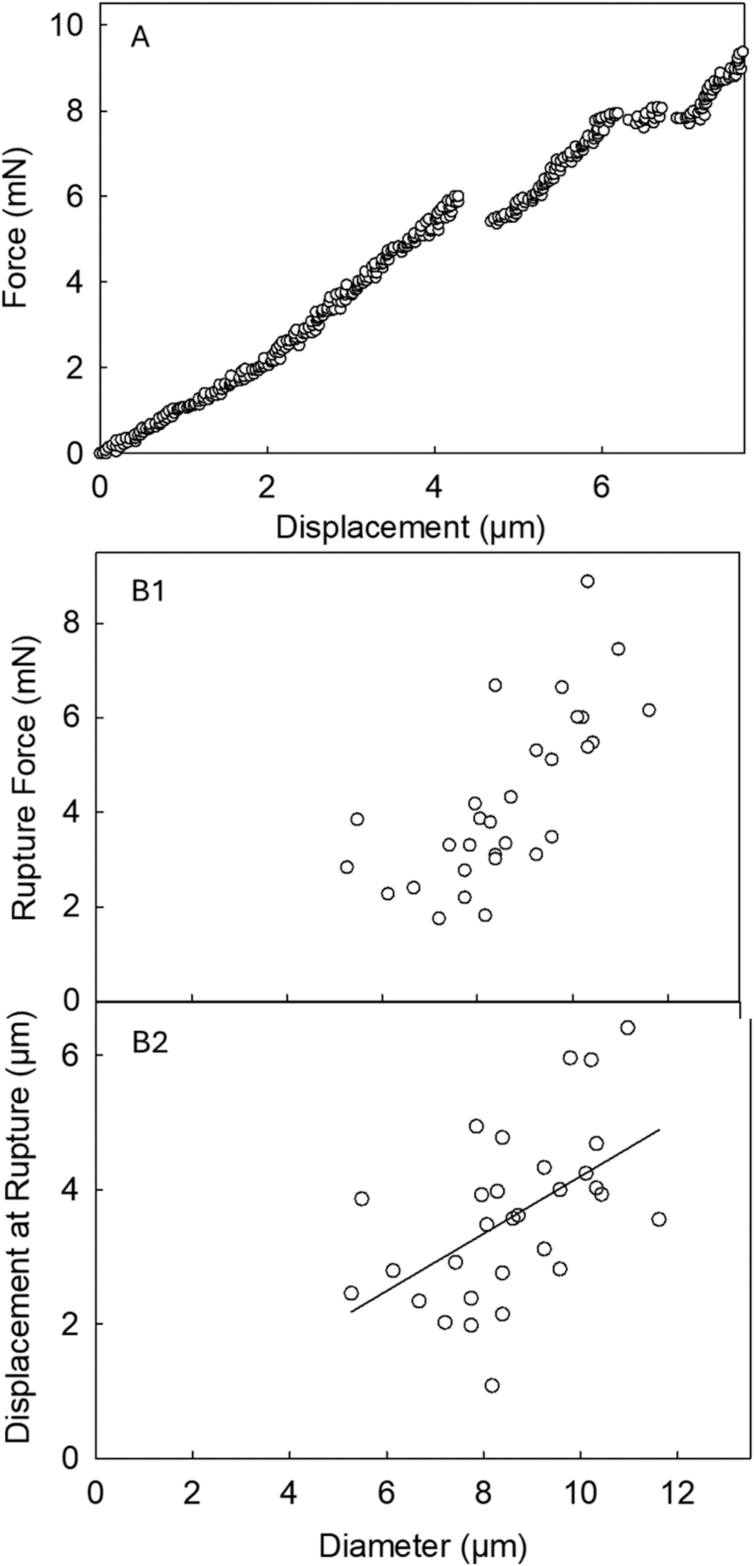
(A) Typical force–displacement curve of composite SiO_2_–PAA-CaCO_3_ (O) microcapsules; Key mechanical property behaviors of composite (O) microcapsules versus their diameter: rupture force (B1) and displacement at rupture (B2).

Figure 11 displays the typical force–displacement curve for composite SiO_2_–PAA-CaCO_3_ microcapsules under compression. The curve is initially monotonically positive, which corresponds to the progressive compression of an individual microcapsule. The compression force associated with the composite microcapsules increased at a specific rate, until a drop in the applied force was observed, indicative of the shell rupture upon compression. Interestingly, the drop was relatively mild, and a subsequent linear-like trend was observed. This was due to the residual mechanical response of the shell post rupture, possibly suggesting the presence of a robust crystalline shell. Such response is typical of particles that catastrophically rupture by cleavage, cracking and splitting under external compression (i.e., from the probe).^55^

Figure 11B1 shows the rupture force of SiO_2_–PAA-CaCO_3_ microcapsules in a dry form as a function of their diameter. There appeared to be a clear increase of the rupture force with diameter, which is in agreement with many other types of microcapsules with similar/different shell chemistries (e.g., chitosan-gum Arabic,^1^ melamine-formaldehyde,^33^ and calcium carbonate-melamine formaldehyde^56^).

Figure 11B2 displays the displacement at rupture of SiO_2_–PAA-CaCO_3_ microcapsules, which also increased with diameter. Their nominal deformation at rupture (derived from the slope of the displacement at rupture vs diameter curve) was relatively large (42 ± 2%) showing that these microcapsules displaced significantly under compression, which is indeed concordant with Figure 11A. This was possibly due to the presence of a rather flexible shell, requiring the probe to travel across a large section of the shell before a cleavage or crack could be induced.

Under compression, the microcapsules yielded a mean nominal rupture stress of 73.5 ± 5.0 MPa and tension of 486 ± 29 N/m (Table 1), which are significantly greater than any microcapsules reported in literature.^56,57^ As a benchmark, the nominal rupture stress of commercial melamine-formaldehyde with similar sizes, for detergent applications, is significantly lower (1.2–1.8 MPa).^57^ Similarly, SiO_2_–PAA-CaCO_3_ microcapsules were also proven much stronger than other PDA-coated CaCO_3_ microcapsules (2.2 ± 0.3 MPa^18^), fabricated without SiO_2_ and/or PAA. This is possibly due to a significantly different shell thickness and/or the presence of SiO_2_ NPs likely acting as an armoring agent. More investigation is required to confirm the reason for this strengthening.

The composite microcapsules possessed a mean number-based diameter 8.7 ± 0.3 μm, which was very similar to the corresponding Sauter diameters (5.4 ± 0.1 μm - 8.8 ± 0.3 μm) obtained by laser diffraction.

For completion, the Hertzian Young’s modulus (*E*_H_) of SiO_2_–PAA-CaCO_3_ microcapsules was quantified based on
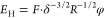
2where *F* is the applied compression force, δ is the indentation (displacement) of the sample at a deformation strain of ∼10%, *R* is the initial radius of the microcapsules, and φ = 9/16 is a coefficient that accounts for the spherical shape inclusive of the Poisson ratio (ν) for incompressible rubber-like matter (ν is taken as 1/2 as the shell materials are assumed to be incompressible, see the comment associated with Supporting Information S8).

It was found that the mean Young’s modulus of composite microcapsules was 869 ± 68 MPa. This shows that the shell of composite microcapsules is significantly stiff and relatively resistant to deformation under compression, as also seen by the large deformation at rupture of 42 ± 2%. This is likely due to the possible presence of a crystalline shell in the composite microcapsules. A typical force versus displacement data fitted by the Hertz model is presented in Supporting Information (Figure S8). For these microcapsules, images were captured during micromanipulation compression (see Supporting Information, Figure S9).

The key mechanical parameters of microcapsules are listed in Table 2, including a comparison with other microcapsules reported in the literature. In our previous work, we fabricated triallyl isocyanurate and mercaptoester (TAIME) microcapsules for phase change materials (PCM), possessing an elastic modulus of 224 ± 7 MPa, which exhibited remarkable stiffness and elastoplastic shell deformation after compression. Nonetheless, the elastic moduli of the microcapsules presented herein are found to be around 3 times higher, which render them potentially suitable for many industrial load-bearing applications, such as in coatings, personal care, or biomedicals.

**Table 2:** Key Intrinsic/Extrinsic Mechanical Property Parameters (Mean ± Standard Error) of Composite Thick-Shell Microcapsules Featuring a SiO_2_–PAA-CaCO_3_ Shell^a^^58^

	SiO_2_-PAA-CaCO_3_	PDA-CaCO_3_	MF-CaCO_3_	TAIME
number-based diameter (μm)	8.7 ± 0.3	30 ± 2	15. ± 1	268 ± 4
rupture force (mN)	4.3 ± 0.3	1.3 ± 0.2	0.09 ± 0.01	142 ± 13
nominal rupture tension (N/m)	486 ± 29	44 ± 6		
nominal rupture stress (MPa)	74 ± 5	2.2 ± 0.3	0.54 ± 0.06	2.5 ± 0.2
displacement at rupture (μm)	3.6 ± 0.2	2.9 ± 0.5	1.1 ± 0.1	161 ± 7
deformation at rupture (%)	42 ± 2	∼10	∼7	∼60
rupture (Yes/No)	yes	yes	yes	yes
elastic modulus (MPa)	869 ± 68			224 ± 7
references	this work	(18)	(13)	(58)

a An equivalent diameter for microcapsules with a slightly elongated morphology was evaluated based on the methodology described in our previous contributions.^17^ Thick-shell microcapsules were assayed for the elastic (Young’s) modulus (coefficient of determination *R*^2^ > 0.9) according to the Hertz model (eq 2). In addition, comparison with other benchmark microcapsules, in the public domain, is provided: (i) PDA-CaCO_3_,^18^ (ii) MF-CaCO_3_,^13^ and (iii) UV-curable polymer constituted of triallyl isocyanurate and mercaptoester (TAIME) for phase change materials (PCM) applications.^58^.

## 4 Conclusions

Environmentally benign core–shell microcapsules with SiO_2_–PAA-CaCO_3_ shells were fabricated, exhibiting a spherical morphology. A polydopamine-based wrapping was subsequently applied, resulting in a smooth bulbous coating. Electron microscopy analysis confirmed the presence of a thick multilayered composite shell (2.4–3.0 μm). FTIR analysis was conducted to investigate the formation of CaCO_3_, tentatively indicating the presence of calcite-based shells. Furthermore, AAS analysis revealed that the chemical bonding due to the interaction between of PAA and calcium ions is relatively strong, yielding a novel pathway-specific binding constant PSBC ∼ 0.63, which is in good agreement with the Ca^2+^-PAA stoichiometric ratio of 0.5. It is likely that the interaction between these two species controls the shell porosity and structural integrity of the resulting microcapsules. Under compression, SiO_2_–PAA-CaCO_3_ microcapsules exhibited a mean nominal rupture stress of 73.5 ± 5.0 MPa and a rupture tension of 485.6 ± 28.9 N/m, outperforming many other microcarriers reported in the literature. In addition, it was found that the Young’s modulus of composite microcapsules was 869 ± 68 MPa, suggesting that these microcapsules possess remarkable stiffness and resistance to deformation, as also seen by the large deformation at rupture of 42.1 ± 2.4%. Within a hydro-propanol receptor medium, the PDA outer layer reduced leakage by approximately 10% in the short term (∼2 h), while the full load was released after 3 days. Overall, our findings suggest that these hazard-free microcapsules offer tunable release and enhanced mechanical properties, opening potential avenues for multiple real-world settings and industrial applications. Scaling up the manufacturing of the microcapsules may be feasible, as the process involves readily available and relatively inexpensive materials, along with standard processing techniques. However, optimization of reaction parameters, cost-effectiveness, and process consistency would need to be thoroughly addressed. Moreover, future research shall be centered on evaluating the biocompatibility and biodegradability of these microcapsules, to meet the demands of ever-evolving fast-paced consumer demand.

## Supporting Information Available

ζ-Potential of SiO_2_ nanoparticles and electrokinetic potential of CTAB, PAA, and combinations; electrokinetic potential of aqueous PAA vs pH, highlighting silica’s pH “comfort zone”; EDX analysis of smooth areas in SiO_2_–PAA-CaCO_3_ microcapsules and corresponding spectra; summary of EDX results for smooth microcapsule areas; EDX analysis of roughened.SiO_2_–PAA-CaCO_3_ microcapsule surfaces; summary of EDX results for coarse microcapsule areas; EDX analysis of PDA-wrapped SiO_2_–PAA-CaCO_3_ microcapsules post-SEM-radiation; EDX results for bulbous and coarse microcapsule areas; cryogenic SEM of microcapsules before and after FIB exposure; EDX elemental mapping for Figure S5; optical microscopy image of irregular Ca-PAA microcapsule clusters; force–displacement data of a microcapsule fitted by the Hertz model; real-time images of composite microcapsules before and after compression (PDF)
