# Supplementary material for: Organic–Inorganic Multilayer Microcarriers with Superior Mechanical Properties for Potential Active Delivery in Fast-Moving Consumer Goods
Source: Ind Eng Chem Res. 2025 Feb 20;64(9):4917–31. doi: 10.1021/acs.iecr.4c04503 (PMC11891905; doi:10.1021/acs.iecr.4c04503)
Supplement: Supplementary file 1 — ie4c04503_si_001.pdf [file ie4c04503_si_001.pdf]

Organic-Inorganic Multi-layer Microcarriers with Superior Mechanical Properties for Potential Active Delivery in Fast-Moving Consumer Goods

Daniele Baiocco[1], Benjamin T. Lobel[2,3], Mohammed Al-Sharabi[4], Olivier J. Cayre[2], Alexander F. Routh[4] and Zhibing Zhang[1*]
[1]School of Chemical Engineering, University of Birmingham, Birmingham, UK
[2]School of Chemical and Process Engineering, University of Leeds, Leeds LS2 9JT, UK
[3]School of Mathematics, Statistics, Chemistry and Physics, Murdoch University, Western Australia, 6150, Australia
[4]Department of Chemical Engineering and Biotechnology, University of Cambridge, Cambridge CB3 0AS, UK
*e-mail: z.zhang@bham.ac.uk

Supporting Information

Contains:

Table S1 - Zeta potential of SiO_2 nanoparticles and electrokinetic potential of CTAB, PAA, and combinations.

Figure S1 - Electrokinetic potential of aqueous PAA vs pH, highlighting silica's pH 'comfort zone'

Figure S2 - EDX analysis of smooth areas in SiO_2-PAA-$CaCO_3$ microcapsules and corresponding spectra.

Table S2 - Summary of EDX results for smooth microcapsule areas.

Figure S3 - EDX analysis of roughened SiO_2-PAA-$CaCO_3$ microcapsule surfaces.

Table S3 - Summary of EDX results for coarse microcapsule areas.

Figure S4 - EDX analysis of PDA-wrapped SiO_2-PAA-$CaCO_3$ microcapsules post-SEM-radiation.

Table S4 - EDX results for bulbous and coarse microcapsule areas.

Figure S5 - Cryogenic SEM of microcapsules before and after FIB exposure.

Figure S6 - EDX elemental mapping for Figure S5.

Figure S7 - Optical microscopy image of irregular Ca-PAA microcapsule clusters.

Figure S8 - Force-displacement data of a microcapsule fitted by the Hertz model.

Figure S9 - Real-time images of composite microcapsules before and after compression.

Table S1 – Zeta potential of SiO_2 nanoparticles (NPs), and electrokinetic potential of cetyltrimethylammonium bromide (CTAB), polyacrylic acid (PAA), and combinations thereof, as used in formulation.

	Electrokinetic potential (mV)
SiO_2 NPs	-31.9 ± 0.1
CTAB	+64.0 ± 0.2
PAA	See Figure S1
SiO_2 NPs - CTAB	+34.1 ± 0.1

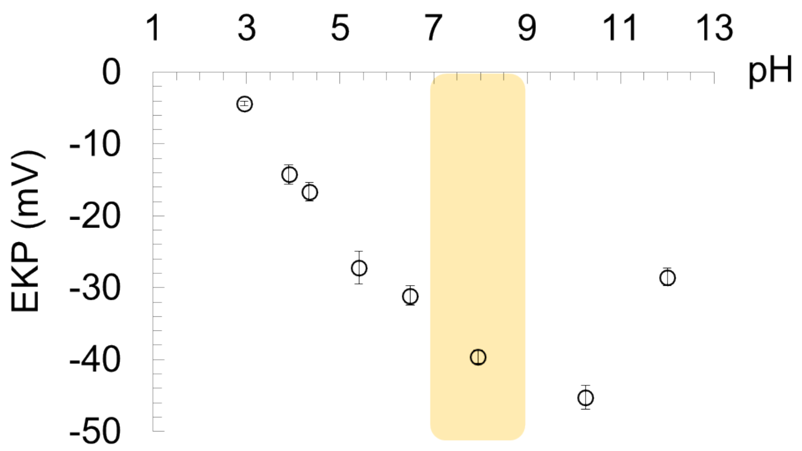

Figure S1 – Electrokinetic potential of aqueous polyacrylic acid versus pH measured by Zetasizer Ultra (Malvern Instruments, UK). The yellow strip identifies the region in which the electrostatic strength of PAA is maximised within the pH 'comfort zone' of silica (the solubility of SiO_2 increases dramatically from approximately pH 9.5).

Comment: pH changes affect the dissociation of carboxyl groups in the polymer chains, leading to variations in the ionisation of polyacrylic acid (PAA) macromolecules. The pK of PAA is ~4.5, depending on its molar mass. This means that below this pH, the undissociated carboxyl groups (–COOH) predominate over the dissociated ones ($–COO^-$). At pH 4.5, the concentrations of dissociated and undissociated carboxyl groups are equal. As the pH increases beyond 4.5, the remaining carboxyl groups progressively dissociate. By pH 6, the PAA macromolecules are almost fully ionised, with a degree of dissociation (α) of 0.97. [26,27]

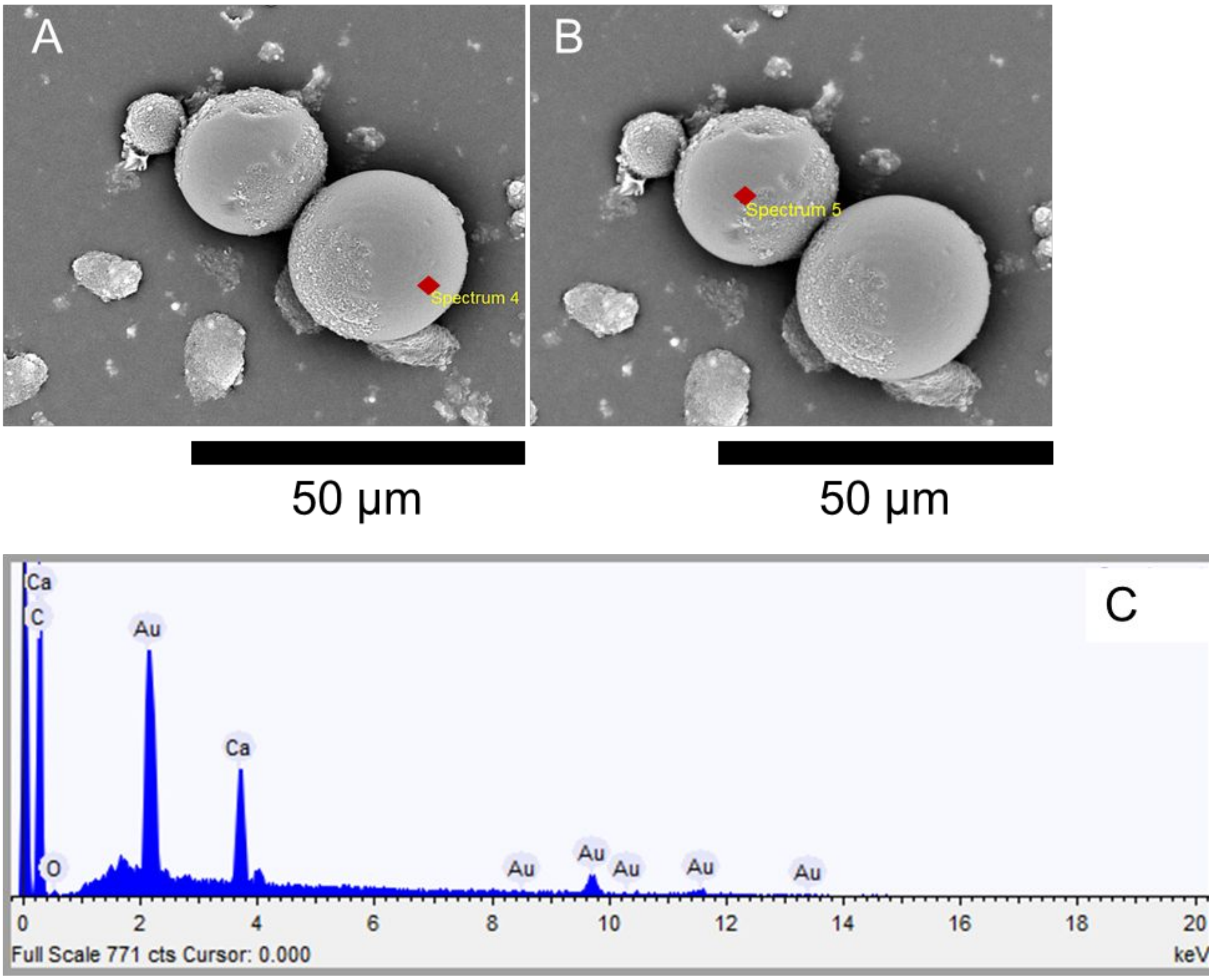

Figure S2 – Energy Dispersive X-Ray (EDX) analysis of SiO_2: (A,B) smooth areas of two individual, fully sealed SiO_2-PAA-$CaCO_3$ microcapsules and (C) typical corresponding EDX spectrum associated with Figure 3D. Observation conditions: Accelerating Voltage = 15 kV, Magnification = x2500, Working Distance = 8.3 mm, Emission Current = 62000 nA, Filament Current=1850 mA.

Table S2 – Summary results of EDX analysis associated with the smooth areas of microcapsules in Figure 3D. A quantitative range of the elemental atomic percentage is provided. **Bold** and underlined values refer to 'Spectrum 4' (A) and 'Spectrum 5' (B), respectively.

Element	Atomic %
Carbon	**88.9** - 92.5
Oxygen	**1.9** - 5.1
Calcium	1.2 - **5.4**
Gold	1.2 - **3.8**

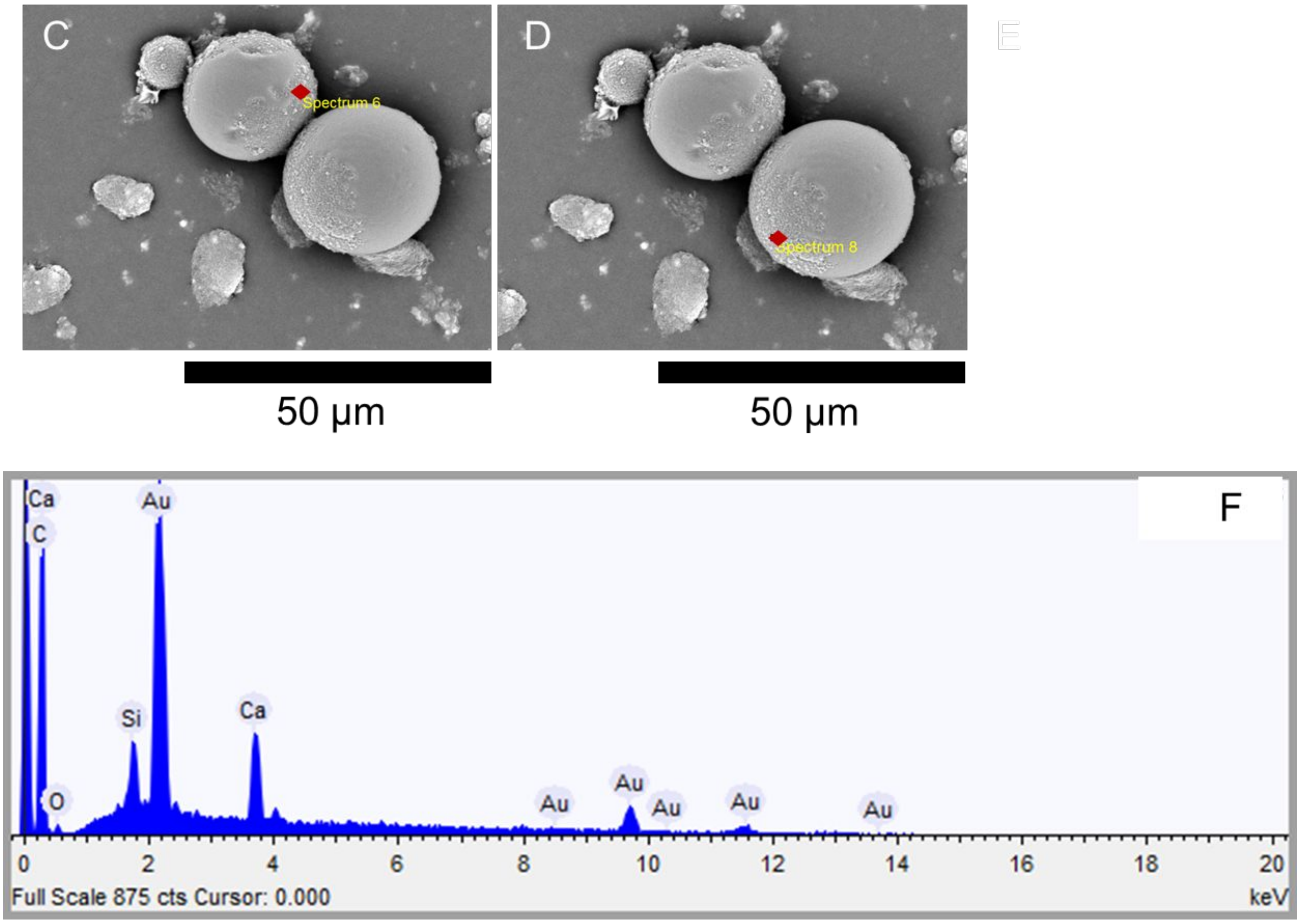

Figure S3 – EDX analysis of: (C-D) roughened surface areas of individual SiO_2-PAA-$CaCO_3$ microcapsules and (F) typical corresponding EDX spectrum associated with Figure 3D.

Table S3 – Summary results of EDX analysis associated with the coarse areas of microcapsules in Figure 3D. A quantitative range of the elemental atomic percentage is provided. **Bold** and underlined values refer to 'Spectrum 6' (C) and 'Spectrum 8' (D), respectively.

Element	Atomic %
Carbon	86.7 - **87.3**
Oxygen	**2.7** - 9.5
Silicon	1.1 - **1.5**
Calcium	1.0 - **3.9**
Gold	1.7 - **4.6**

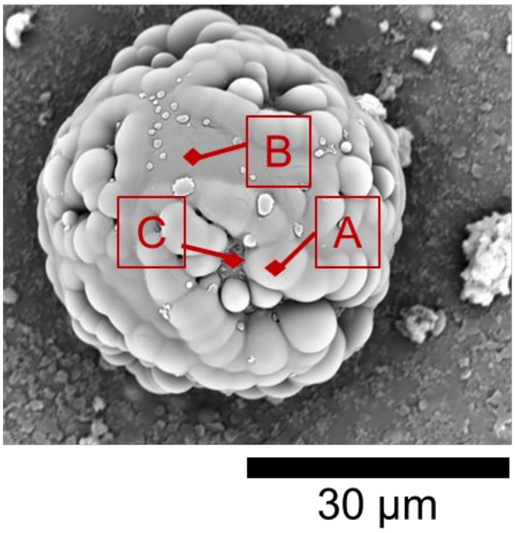

Figure S4 – EDX analysis of PDA-wrapped SiO_2-PAA-$CaCO_3$ microcapsules following SEM-radiation (A, B) and (C) beneath the PDA wrapping. A quantitative range of the elemental atomic percentage is provided. **Bold** and underlined values refer to 'Spectrum 2' (A) and 'Spectrum 1' (B), respectively. N.D. = not detected.

Table S4 – Summary results of EDX analysis associated with the bulbous (PDA-wrapped, (A,B)) and coarse (Spectrum 4' (C)). areas of microcapsules in Figure 4. A quantitative range of the elemental atomic percentage is provided. **Bold** and underlined values refer to 'Spectrum 2' (A), 'Spectrum 1' (B), respectively.

Element	Atomic (%) (A, B)	Atomic (%) (C)
Carbon	91.4 - **93.1**	88.7
Oxygen	**3.0** - 5.9	N.D
Silicon	N.D.	1.1
Calcium	1.4 - **2.3**	7.8
Gold	1.3 - **1.6**	2.5

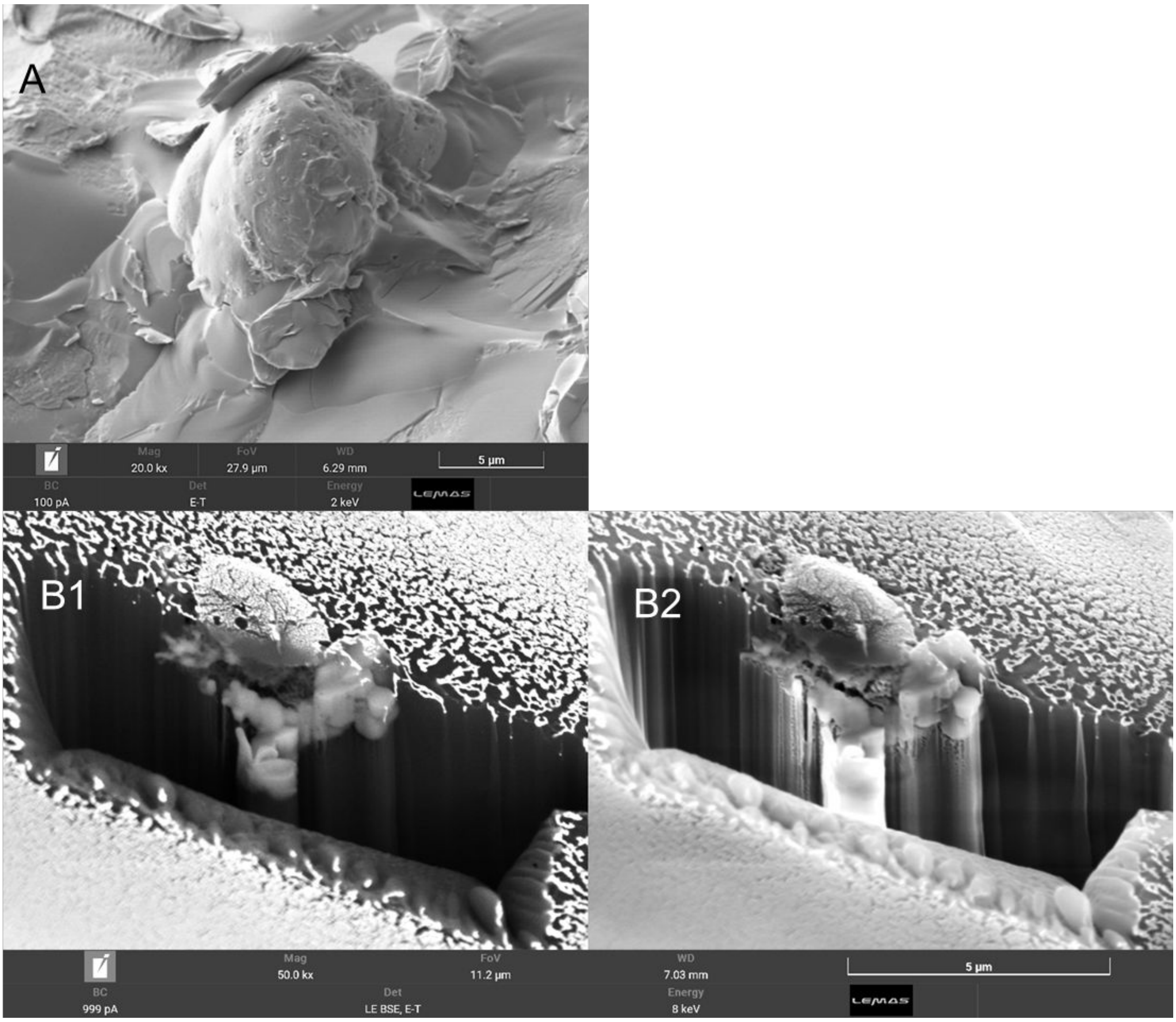

Figure S5 – Cryogenic SEM micrographs of microcapsule (A) before and (B1, B2) after exposure to focused ionised beam (FIB; beam current 999 pA; working distance ~ 7.03 mm; energy 8 keV).

Comment: The microcapsule appears relatively spherical, featuring several irregularly shaped protrusions. The surface is rather smooth, though it exhibits localised veining, which may suggest a complex heterogeneous composition with jagged edges. These features could be attributed to differential layers or inclusions, primarily composed of silica, calcium, and carbon-based ligands (see Figure S6).

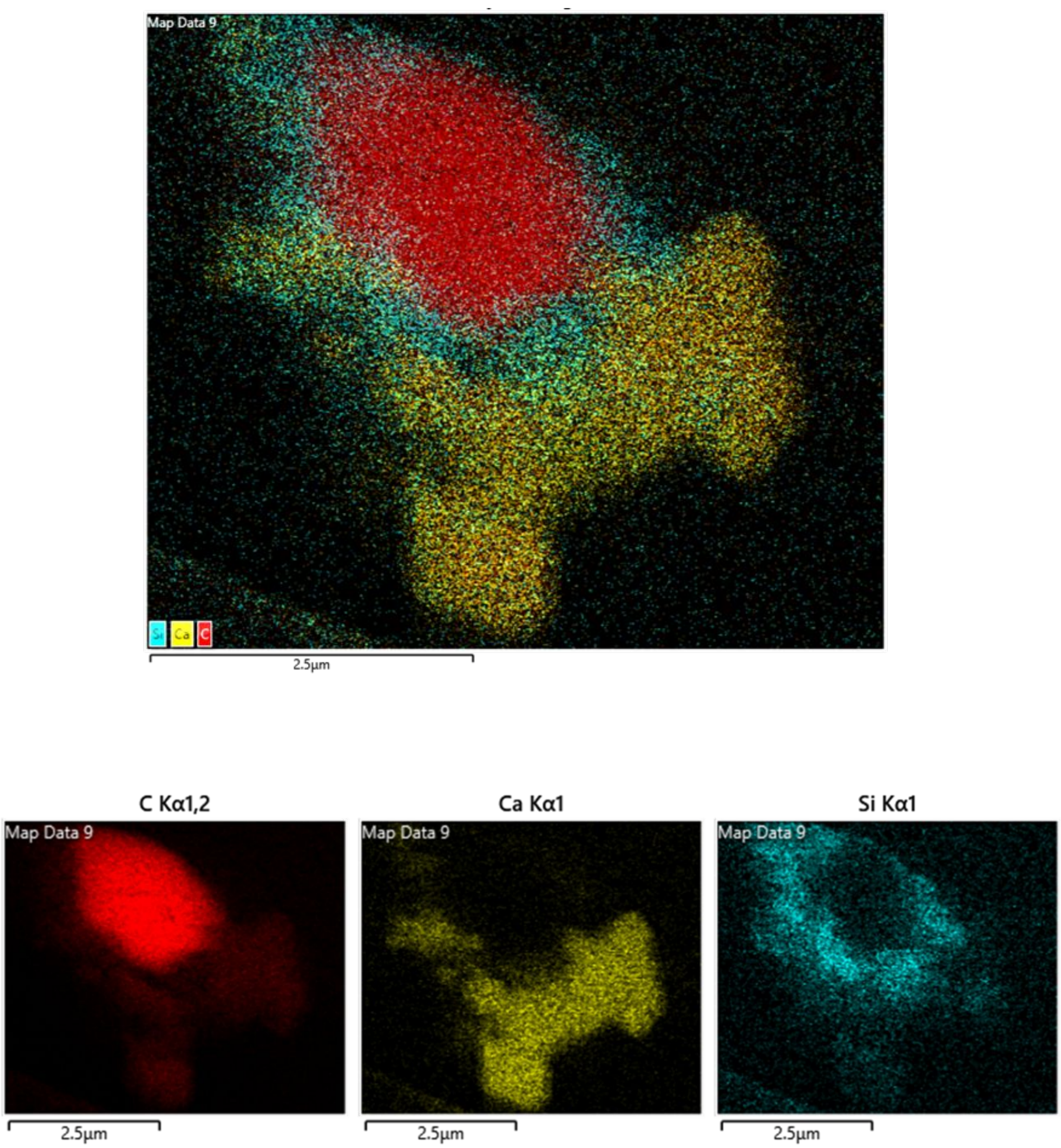

Figure S6 – Elemental detection by EDX layered mapping of Figure S5 (Carbon (C) in red, Calcium (Ca) in yellow, Silicon (Si) in light blue).

Comment: The innermost structure resembles a round core, which is indeed carbon-based (e.g., hexyl salicylate). These observations seem to support the core-shell structure of microcapsules.

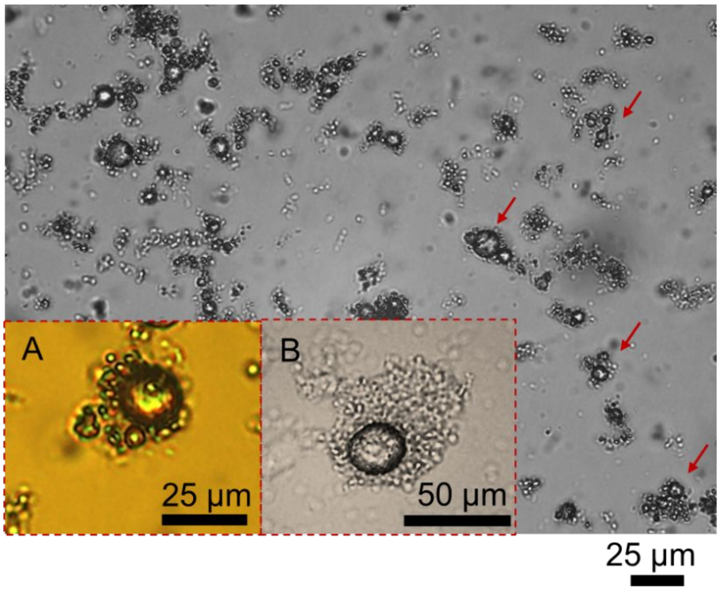

Figure S7 – Optical microscopy image of Ca-PAA microcapsules forming irregularly shaped clusters (see red arrows and insets A and B).

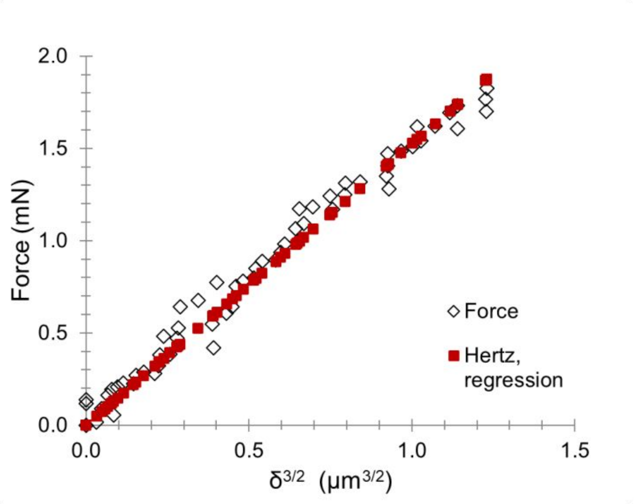

Figure S8 – Typical force (F) versus displacement (δ) data of a microcapsule (diameter ~ 11.6 μm; Young's modulus, E ~ 1.01 GPa) fitted by the Hertz model (coefficient of determination R^2 ~ 0.98).

Comment: Core-shell microcapsules consist of a relatively large liquid core and relatively thin solid shell, the former of which is incompressible. The Poisson ratio of calcium carbonate (calcite crystals) is in a range of 0.30-0.33, suggesting a relative ductility of the crystals. However, the effect of Poisson ratio on the calculated value of Young's modulus is small (less than 20% between the Poisson ratio of 0.32 and 0.5). For simplicity, we have assumed the whole microcapsules to be incompressible, i.e. Poisson ratio equals 0.5, which is a reasonably good approximation.

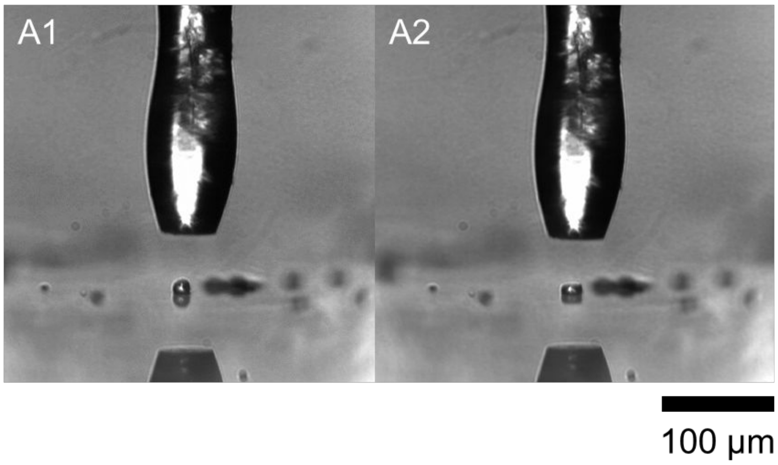

Figure S9 – Real-time side-view images of composite thick-shell microcapsules (~12 µm) before (A1) and after (A2) compression by micromanipulation.
